# Cofilin-1 Is a Mechanosensitive Regulator of Transcription

**DOI:** 10.3389/fcell.2020.00678

**Published:** 2020-07-30

**Authors:** Catarina Domingues, A. Margarida Geraldo, Sandra Isabel Anjo, André Matos, Cláudio Almeida, Inês Caramelo, José A. Lopes-da-Silva, Artur Paiva, João Carvalho, Ricardo Pires das Neves, Bruno Manadas, Mário Grãos

**Affiliations:** ^1^Center for Neuroscience and Cell Biology (CNC), University of Coimbra, Coimbra, Portugal; ^2^Institute for Interdisciplinary Research, University of Coimbra (IIIUC), Coimbra, Portugal; ^3^Polytechnic Institute of Coimbra, Coimbra College of Agriculture, Coimbra, Portugal; ^4^LAQV-REQUIMTE, Department of Chemistry, University of Aveiro, Aveiro, Portugal; ^5^Flow Cytometry Unit, Department of Clinical Pathology, Centro Hospitalar e Universitário de Coimbra, Coimbra, Portugal; ^6^Coimbra Institute for Clinical and Biomedical Research, Faculty of Medicine, University of Coimbra, Coimbra, Portugal; ^7^Center for Innovative Biomedicine and Biotechnology, University of Coimbra, Coimbra, Portugal; ^8^Instituto Politécnico de Coimbra, ESTESC-Coimbra Health School, Ciências Biomédicas Laboratoriais, Coimbra, Portugal; ^9^Centro de Física da Universidade de Coimbra (CFisUC), Department of Physics, University of Coimbra, Coimbra, Portugal; ^10^Biocant, Technology Transfer Association, Cantanhede, Portugal

**Keywords:** mechanotransduction, Cofilin-1, cytoskeleton, transcription, hUCM-MSCs, proteomics

## Abstract

The mechanical properties of the extracellular environment are interrogated by cells and integrated through mechanotransduction. Many cellular processes depend on actomyosin-dependent contractility, which is influenced by the microenvironment’s stiffness. Here, we explored the influence of substrate stiffness on the proteome of proliferating undifferentiated human umbilical cord-matrix mesenchymal stem/stromal cells. The relative abundance of several proteins changed significantly by expanding cells on soft (∼3 kPa) or stiff substrates (GPa). Many such proteins are associated with the regulation of the actin cytoskeleton, a major player of mechanotransduction and cell physiology in response to mechanical cues. Specifically, Cofilin-1 levels were elevated in cells cultured on soft comparing with stiff substrates. Furthermore, Cofilin-1 was de-phosphorylated (active) and present in the nuclei of cells kept on soft substrates, in contrast with phosphorylated (inactive) and widespread distribution in cells on stiff. Soft substrates promoted Cofilin-1-dependent increased RNA transcription and faster RNA polymerase II-mediated transcription elongation. Cofilin-1 is part of a novel mechanism linking mechanotransduction and transcription.

## Introduction

Cells sense and respond to the mechanical properties of the extracellular environment. Specifically, mechanotransduction originated at the cell-extracellular matrix (ECM) interface (or cell-substrate interface in case of *in vitro* cell culture) initiates at the focal adhesions (FAs). FAs encompass several proteins like integrins (transmembrane receptors that bind to EMC proteins, constituting anchoring points of adherent cells), adapter proteins like talin and vinculin (bridging integrins with the actin cytoskeleton), as well as signalling proteins like focal adhesion kinase (FAK) (reviewed in Vining and Mooney, 2017). Upon activation of integrins, these proteins interact with each other, leading to the formation of FAs and subsequent recruitment of the actin cytoskeleton (Humphries et al., 2007; Thievessen et al., 2013). The formation of FAs leads to the activation of a wide range of signalling pathways, several of which converge on and activate RhoA, a member of the Rho GTPase family (Lessey et al., 2012). In turn, active RhoA engages its downstream effector Rho-associated protein kinase (ROCK), and subsequently, the motor protein non-muscle myosin-II (NMM-II) (Marjoram et al., 2014; Burridge et al., 2019). Activation of NMM-II leads to the contraction of actin stress fibres, constituted by crosslinked anti-parallel filamentous (*F*)-actin bundles, resulting in the build-up of intracellular tension. Hence, intracellular tension occurs as the result of the contractile forces generated by actomyosin spanning from the plasma membrane (at FAs) to the nucleus (through the linker of nucleoskeleton and cytoskeleton — LINC — complex) (Jaalouk and Lammerding, 2009; Burridge and Guilluy, 2016), which dictates to a great extent the mechanical properties of the cell. If the stiffness of the extracellular matrix (or substrate if *in vitro*) is high, the reinforcement of FAs occurs, resulting in increased intracellular contractility and mechanical stress exerted on the ECM and the nucleus (Moore et al., 2010; Klapholz and Brown, 2017). Hence, cells probe the ECM by exerting forces (intrinsic forces), subsequently responding according to the mechanical properties of the environment. Likewise, cells also sense and respond to forces originating on the ECM/substrate (extrinsic forces). The intrinsic or extrinsic forces occurring as the result of actomyosin contractility as a function of ECM/substrate stiffness, or other mechanical cues provided by the microenvironment influence many aspects of cell biology, including proliferation, differentiation and gene expression (Sun et al., 2012; Discher et al., 2017; Kumar et al., 2017; Uhler and Shivashankar, 2017; Vining and Mooney, 2017).

Another consequence of the activation of RhoA and ROCK is the stabilisation of F-actin by a mechanism involving LIM Kinase 1 (LIMK1), which in turn phosphorylates and inhibits Cofilin-1 (Yang et al., 1998; Maekawa et al., 1999; Mizuno, 2013; Prunier et al., 2017). Cofilin-1 is an essential actin-regulating protein, promoting the severing of actin filaments and disassembly of F-actin into globular actin (G-actin), playing a central role in actin cytoskeleton dynamics (Bamburg and Bernstein, 2010; Ohashi, 2015). Interestingly, both RhoA and ROCK activity and F-actin/G-actin ratio increase with substrate stiffness (Engler et al., 2006; Fu et al., 2010; Trichet et al., 2012; Gupta et al., 2015; Gerardo et al., 2019). The activity and subcellular localisation of Cofilin-1 depend on the phosphorylation state of its Ser3, a target of LIMK1. Phosphorylated Cofilin-1 is inactive and remains in the cytosol, while the non-phosphorylated form is active and able to severe and depolymerise F-actin (Nebl et al., 1996; Yang et al., 1998), adopting a subsequent nuclear localisation (Munsie et al., 2012). Other important cellular functions have been described for Cofilin-1. It is required for the nuclear transport of G-actin (through a mechanism mediated by importin 9) (Dopie et al., 2012; Percipalle, 2013) and together promote transcription elongation mediated by RNA polymerase II (Obrdlik and Percipalle, 2011). More recently, Cofilin-1 was identified as an essential protein for normal nuclear structure and function in distinct cell types (Wiggan et al., 2017).

In this study, we sought to explore how the proteome of human mesenchymal stem/stromal cells obtained from the umbilical cord matrix (hUCM-MSCs) is regulated when cells are cultured on substrates with distinct stiffness. There are two reasons for using this cellular model. First, MSCs are highly mechanosensitive and have been used extensively for studies in the field of mechanobiology (Engler et al., 2006; Fu et al., 2010; Yang et al., 2014; Gerardo et al., 2019). Second, MSCs are very promising for clinical applications, but require extensive *in vitro* expansion before being used. It is known that *in vitro* expansion of MSCs using standard cell-culture conditions leads to the loss of cell potency (Hoch and Leach, 2014; Müller et al., 2015; Galipeau et al., 2016). Increasing evidence suggests that the high stiffness of substrates typically used in standard cell culture conditions (like for example tissue culture polystyrene — TCPS —, with Young’s modulus in the GPa range), several orders of magnitude higher than the natural cellular microenvironment, contributes significantly to such loss (Lee et al., 2014; Yang et al., 2014; Kusuma et al., 2017). Interestingly, a recent report indicates that the proliferative and differentiation potential of MSCs (at least toward the adipogenic lineage) is prolonged and senescence is delayed when cells are extensively cultured on a polyacrylamide 5 kPa substrate in comparison with stiff TCPS, while maintaining typical MSC surface markers (Kureel et al., 2019). Although in a distinct context, our studies also indicate that MSC stemness is favoured by soft substrates (Gerardo et al., 2019).

MSCs are a heterogeneous population of stromal cells, including multipotent adult stem cells, that can be isolated from vascularised tissues like bone marrow, adipose tissue and umbilical cord (Wagner et al., 2005). MSCs possess well-known cell-surface markers (positive expression of CD105, CD90, CD73 and negative expression of haematopoietic markers like CD34, CD45, HLA-DR, CD14 or CD11B, CD79α or CD19) and can differentiate *in vitro* into osteoblasts, chondroblasts and adipocytes (Pittenger et al., 1999; Dominici et al., 2006). MSCs can migrate *in vivo* to damaged tissues in response to cytokines/chemokines, growth-factors or adhesion molecules and therein provide potent immunomodulatory and regenerative responses (Kim and Cho, 2013). Also, MSCs have been extensively used for cell-based therapies in clinical trials (Squillaro et al., 2016). Furthermore, it was recently reported that MSCs retain mechanical information from their past physical extracellular environment, developing mechanical memory that is dependent on the time in culture, or mechanical dosing. This has significant implications in stem cell function and differentiation (Yang et al., 2014; Peng et al., 2017), reinforcing the need to develop new strategies for stem cell maintenance and expansion *in vitro*. Currently, a variety of substrates that mimic distinct aspects of the ECM are available for cell culture, providing cell adhesion and mechanical support (Green and Elisseeff, 2016). The influence of mechanical and biochemical properties of such ECM-like substrates for the differentiation of MSCs into distinct lineages has been studied for a decade now (Engler et al., 2006; Fu et al., 2010). However, the effect of biophysical elements on proliferating undifferentiated MSCs is largely unknown and remains to be studied employing biologically relevant and comprehensive approaches.

This study presents a comparative and quantitative proteomics analysis of hUCM-MSCs cultured on stiff TCPS and soft polydimethylsiloxane (PDMS) substrates, which allowed the identification and characterisation of Cofilin-1 as a mechanosensitive protein involved in the regulation of transcription in response to substrate stiffness. To our knowledge, this is the first report of a quantitative and comprehensive characterisation of the MSC proteome in the mechanobiology field, with an expected impact on future studies evaluating MSCs’ therapeutic effectiveness and clinical value. The link between mechanotransduction, Cofilin-1 and transcription is also novel, which will open a new research avenue regarding the regulation of gene expression upon modulation by mechanical factors.

## Materials and Methods

### Cell Culture

Human umbilical cords were obtained after birth from healthy donors upon informed consent from the parent(s) and the study was approved by the Ethics Committee of the Faculty of Medicine, University of Coimbra, Portugal (ref. CE-075/2019). All methods were carried out in accordance with national and European guidelines and regulations. MSCs were isolated from cryopreserved fragments of human umbilical cord matrix (UCM) as described (Leite et al., 2014) with some modifications, as indicated. Briefly, cryopreserved fragments from human UCM were thawed at 37°C and washed with Alpha-MEM medium supplemented with 100 U/ml of Penicillin, 10 μg/ml Streptomycin and 2.5 μg/ml Amphotericin B (all from Life Technologies). Groups of 30 fragments were transferred to 21 cm^2^ TCPS (Corning Costar) and left to dry for 30 min. Then, the proliferation medium [Alpha-MEM with 10% (v/v) MSC-qualified foetal bovine serum (FBS) (Hyclone, GE Healthcare) and antibiotics (as above)] was added to the culture plates until the fragments were immersed. Plates were incubated at 37°C with 5% CO_2_/95% air and 90% humidity until the formation of well-defined MSC colonies was observed by phase-contrast microscopy. Next, fragments were removed, and adherent cells were detached using Trypsin (500 μg/ml)-EDTA (200 μg/ml) solution (Life Technologies) and re-seeded on a new plate (passage 1 – P1) in order to homogenise the culture. hUCM-MSCs were expanded in TCPS until P1 and then seeded and maintained on the distinct cell culture substrates between P2 and P4. For proliferation kinetics experiments, cells were passaged until P6.

The human foetal lung fibroblast cell line (MRC-5) (Steurer et al., 2018) was cultured in DMEM/F12 with 10% (v/v) FBS (both from Life Technologies) and antibiotics (as above).

### Flow Cytometry

Immunophenotypic characterisation of hUCM-MSCs was performed at P4 for four independent samples. Cells were dissociated using Accutase (LifeTechnologies) and then stained with the monoclonal antibodies (mAbs) for surface antigens during 30 min in the dark, at room temperature (RT). The mAb panel used for the characterisation of hUCM-MSCs is detailed in Supplementary Table S1. For all mAbs, we used the concentration recommended by the manufacturer. Then, cells were washed and resuspended in PBS, and immediately acquired in a FACSCanto^TM^II (BD) flow cytometer equipped with the FACSDiva software (v6.1.2; BD). Data analysis was performed using the Infinicyt software (version 1.7; Cytognos SL, Salamanca, Spain).

### Proliferation Kinetics

hUCM-MSCs were cultured on TCPS and 40:1 PDMS at 3,000 cell/cm^2^ from P2 to P6. Cells were counted once they reached 80% confluence at each passage and the following parameters were calculated (Leite et al., 2014): total numbers of cells, population doubling, cumulative population doubling and generation time. Total numbers of cells was calculated using the formula *TNC* = *N*_*H*_×*B*/*N*_*I*_, in which *N*_*H*_ represents the number of cells harvested at the end of each passage, *B* represents the total number of cells from the previous passage and *N*_*I*_the number of cells plated in each passage. Population doubling rate was calculated using the equation *N*_*H*_/*N*_*I*_ = 2^*PD*^ or *PD* = [*log*_10_⁡(*N*_*H*_)−*log*_10_⁡(*N*_*I*_)]/*log*_10_⁡(2). The population doubling for each passage was calculated and added to that of the previous passages to obtain cumulative population doubling. The generation time – average time between two cell doublings – was calculated from P3 to P6 using the following formula: *GT* = [*log*_10_⁡(2)×Δ*t*]/[*log*_10_⁡(*N*_*H*_)−*log*_10_⁡(*N*_*I*_)].

### Pharmacological Treatments

For pharmacological treatments, hUCM-MSCs were seeded at 7,000 cells/cm^2^ on coated custom-made 40:1 PDMS, glass coverslips or TCPS. Twenty four hour after seeding, cells on soft PDMS were incubated with 25 μM of lysophosphatidic acid (LPA – Enzo, BML-LP100) for 2 h. Cells on glass coverslips or TCPS were incubated with 30 or 50 μM of racemic Blebbistatin (Calbiochem, 203389-5MG), respectively, for 24 h. Cells cultured on TCPS were incubated with 10 or 12 μM of LIM Kinase inhibitor (Limki-3 – Calbiochem, 435930) for 24 h. Stock solutions were prepared in dimethyl sulfoxide (DMSO, Santa Cruz Biotechnology) and the incubations were performed in a serum-free medium. For MRC-5 cells the procedures were similar. Briefly, cells were cultured at 4,000 cells/cm^2^ on glass coverslips or 1.5 kPa PDMS (IBIDI). Cells on glass coverslips were cultured for 32 h and then incubated with 25 μM of Blebbistatin for an additional 40 h. Cells on PDMS were cultured for 70 h and then incubated with 1 μM of LPA for 2 h. In both cases, cells were fixed after 72 h in cultured and immunostained as indicated.

### Preparation of Cell Culture Substrates

The substrates used for cell culture included TCPS (Corning Costar), glass coverslips (Thermo Fischer Scientific) and PDMS (1.5 and 15 kPa μ-Dish from IBIDI and ∼3 kPa custom-made substrates).

Custom-made PDMS substrates were prepared by mixing the silicone elastomer base with the curing agent (Sylgard 184; Dow Corning/Dowsil) on a 40:1 proportion. In order to remove air bubbles, the mixture was degassed under vacuum (−5 inHg) at RT for 40 min. Next, a specific volume of that mixture was poured according to the area of each platform used in order to create substrates approximately 300 μm thick (higher than the recommended substrate thickness to avoid cells from sensing the underlying stiff support) (Buxboim et al., 2010). PDMS substrates were then cured at 80°C for 4 h in an incubator (Memmert).

Since PDMS is highly hydrophobic and does not readily bind to cell-adhesion proteins, all PDMS substrates were chemically treated (based on existing literature; Yu et al., 2009; Kuddannaya et al., 2013) in order to become more hydrophilic and receptive to protein coating and cell culture as described before (Gerardo et al., 2019). Solution 1 [double deionised (dd) H_2_O, 37% hydrochloric acid (Fluka) and 30% (w/w) hydrogen peroxide (Sigma) in a volumetric proportion of 5:1:1] was added over the PDMS surface for 5 min at RT. Substrates were then washed three times with abundant ddH_2_O and treated with Solution 2 [10% (v/v) of 3-aminopropyltrimethoxisilane (3-APTMS, Alfa Aesar) in 96% ethanol (Merck)] for 30 min at RT. Next, the substrates were washed three times with ddH_2_O (10 min each) with agitation. Finally, the substrates were incubated with Solution 3 [3% (w/v) glutaraldehyde in PBS] for 20 min at RT followed by three washes with ddH_2_O (5 min each) with agitation. Glass coverslips were treated as described for PDMS except that 1M NaOH (Merck) was used (30 min with agitation) instead of Solution 1. After functionalisation, PDMS substrates and glass coverslips were exposed to ultraviolet light for 30 min in an air flow cabinet for sterilisation.

To allow cell adhesion, the surface of PDMS substrates and glass coverslips were coated with human plasma purified fibronectin (FN) (Merck Millipore) and rat tail type-I collagen (COL-I) (BD Biosciences) in PBS at a final concentration of 10 and 17 μg/ml, respectively. The coating solution was used at 143 μl/cm^2^, resulting in 1.4 μg/cm^2^ of FN and 2.4 μg/cm^2^ of COL-I. All substrates were incubated with the coating solution for 4 h at 37°C, and then washed once with sterile PBS before cell seeding. The TCPS dishes used in this study were not coated.

### Characterisation of PDMS Substrates

The rheological characterisation of custom-made 40:1 PDMS substrates was performed by small-strain oscillatory shear tests using a Kinexus Pro rheometer and rSpace software (Malvern) fitted with a parallel plate geometry (stainless steel wrinkled plate, 4 cm diameter). Frequency sweeps were performed from 10 to 0.1 Hz (five reads per decade) with 1% strain at 37°C and under a normal force of 0.5 N to guarantee adherence. The Young’s modulus (*E*) was calculated using the values measured for viscoelastic shear modulus and using the formula E = 2*G*′(1+ν), in which *G*′ is the shear storage modulus at 1 Hz and ν the Poisson’s ratio, assumed to be 0.5 as for materials whose volume do not change upon stretching.

To measure the thickness of the PDMS substrates, a pre-polymer to curing agent ratio of 10:1 was used, since polymers prepared with this formulation could readily be detached from the dishes in which were cured. After curing (as described above), the substrates were detached from the dishes and sliced across the centre. The thickness of the central zone of the PDMS substrates was measured using a phase-contrast microscope (Axiovert 40C) and the AxioVision software (both from Zeiss).

### Subproteome Fractionation

Subproteome fractionation was performed using hUCM-MSCs at the end of P4 after expansion in TCPS or custom-made PDMS from P2 to P4, using the protocol described in Anjo et al. (2017). Briefly, cells were washed once with PBS and then incubated with extraction buffer [50 mM Tris-HCl pH 7.4 supplemented with protease inhibitors – Protease Inhibitor Cocktail tablets, Complete EDTA-free (Roche)]. Next, cells were subjected to ultrasonication in a H_2_O-bath (Vibra-Cell 750 watts, Sonics) with 40% amplitude and 30 s cycles. After centrifugation (1,000 × *g*) for 5 min at 4°C, supernatants were ultracentrifuged (126,000 × *g*) for 1 h at 4°C (Beckman Coulter), and the pellet corresponding to the membrane-enriched fraction was solubilised in SDS sample buffer [1.7% (w/v) SDS and 100 mM DTT in 50 mM Tris pH 6.8]. Five volumes of cold acetone were added to each supernatant (corresponding to the soluble fraction) and samples were stored at −20°C to precipitate the protein content, which was recovered by centrifugation at 4,000 × *g* during 30 min at 4°C and then the protein pellets were washed with cold acetone. Next, the pellet corresponding to the soluble fraction was resuspended in SDS sample buffer. Protein quantification was performed using the Direct Detect Spectrometer (Millipore) according to the manufacturer’s instructions, and 100 μg of protein (soluble or membrane fraction) were used for sequential windowed data independent acquisition of total high-resolution mass spectra (SWATH-MS) analysis.

### SWATH-MS Analysis

After denaturation at 95°C, samples were alkylated with acrylamide and subjected to in-gel digestion using the short-GeLC approach (Anjo et al., 2015). Pooled samples were created for protein identification and the same amount of MBP-GFP was added to all samples to be used as an internal standard. Samples were analysed on a Triple TOF^TM^ 5600 System (AB Sciex^®^) in two phases: information-dependent acquisition (IDA) of the pooled samples for protein identification and SWATH acquisition of each individual sample for quantification (detailed in the Supplementary Methods). A specific library of precursor masses and fragment ions was created by combining all files from the IDA experiments, and used for subsequent SWATH processing. Libraries were obtained using ProteinPilot^TM^ software (v5.1, AB Sciex^®^) searching against a database composed by *Homo sapiens* from Swiss-Prot and the sequence of the recombinant protein MBP-GFP. SWATH data processing was performed using SWATH^TM^ processing plug-in for PeakView^TM^ (v2.0.01, AB Sciex^®^). Peptides were selected automatically from the library and up to 15 peptides with up to 5 fragment ions were chosen per protein. Quantitation was attempted for all proteins in the library file that were identified below 5% local FDR from ProteinPilot^TM^ searches, by extracting the peak areas of the target fragment ions of those peptides using an extracted-ion chromatogram (XIC) window of 3 and 4 min (for soluble and membrane-enriched fraction, respectively) with 20 ppm XIC width.

All the peptides that met the 1% FDR threshold in at least one sample were retained and the levels of the proteins were estimated by summing the respective transitions and peptides that met the criteria established (an adaptation of Collins et al., 2013). For comparisons between experimental conditions, the protein levels were subjected to two steps of data normalisation: (1) normalised to the internal standard (MBP-GFP) followed by (2) a normalisation using the sample total intensity.

The mass spectrometry proteomics data have been deposited to the ProteomeXchange Consortium via the PRIDE (Vizcaíno et al., 2016) partner repository with the dataset identifier PXD017674.

### Bioinformatics and Data Analysis

PANTHER Classification System was executed for Gene Ontology analysis. Gene Ontology enrichment analysis was performed for proteins identified using the web-based application Gene Ontology enrichment analysis and visualisation tool – GOrilla. In order to identify and collect information about the proteins that were found, UniProt was used. Venn graphs were generated using BioVenn web application.

### Protein Extracts and Western Blot Analysis

To obtain whole-cell protein extracts from hUCM-MSCs (P2–P4), cells were detached using a cell scraper in the presence of Laemmli buffer [120 mM Tris-HCl pH 6.8, 4% SDS (w/v) and 20% glycerol (v/v)], heated for 5 min at 95°C and passed ten times through a 25G needle. Total protein was quantified using Pierce^TM^ 660 nm Protein Assay (Thermo Fischer Scientific) according to manufacturer’s instructions followed by addition of DTT to each protein sample at a final concentration of 0.1 M. Protein extracts (10 or 20 μg) were separated by SDS-PAGE as previously described (Leite et al., 2014) and transferred onto polyvinylidene fluoride (PVDF) membranes (Bio-Rad). The membranes were blocked in TBS- or PBS-0.1% (v/v) Tween 20 containing 5% (w/v) non-fat dried milk for 1 h at RT. Incubations with the antibodies indicated in the Supplementary Table S2 were performed with gentle agitation overnight (ON) at 4°C followed by 1 h at RT. All membranes were washed with TBS- or PBS-0.1% (v/v) Tween 20 and then incubated for 1 h at RT with gentle agitation with the respective alkaline phosphatase-conjugated secondary antibody (Jackson ImmunoResearch) diluted in blocking solution. Next, membranes were incubated with enhanced chemifluorescence (ECF) kit (GE Healthcare) according to the manufacturer’s instructions and detection was performed on a Molecular Imager FX Pro Plus system using the software Quantity One (both from Bio-Rad). To quantify the total protein in each lane, the membranes were stained using SERVA purple (SERVA electrophoresis, Enzo) according to the manufacturer’s guidelines. The integrated density of antibody-stained protein bands and total protein content of each lane were measured using Quantity One software (Bio-Rad).

### Immunocytochemistry, Fluorescence Microscopy and Image Analyses

hUCM-MSCs between P2–P4 on glass coverslips or custom-made PDMS substrates were fixed with 4% (w/v) paraformaldehyde (PFA, Santa Cruz Biotechnology) in PBS for 20 min at RT. Immunocytochemistry (ICC) was performed as detailed in Leite et al. (2014). Fixed cells were washed three times with PBS and permeabilised with 0.1% Triton X-100 in PBS for 20 min. Cells were blocked with 1% (w/v) BSA (Calbiochem) in PBS for 30 min at RT and then incubated with primary antibodies (Supplementary Table S2) diluted in blocking solution ON at 4°C in humidified conditions. Next, cells were washed with PBS and incubated with the appropriate secondary antibodies (Supplementary Table S2) in PBS with 1% (w/v) BSA for 1 h at RT. For nuclear staining, cells were incubated with DAPI (Supplementary Table S2) for 5 min. To visualise polymerised actin, cells were stained with TRITC-labelled Phalloidin (Supplementary Table S2) for 1 h at RT and then washed three times with PBS (5 min each) to remove unbound reagent. Fluorescence microscopy was performed using a Zeiss Axiovert 200M microscope using AxioVision Release 4.8 software (Zeiss). Exposure time was the same for each analysed marker and for each independent experiment. To quantify the mean of fluorescence intensity (MFI) of F-actin, the regions of interest (ROIs) were defined as limiting cells by the edges. DAPI images were used to defined ROIs to measure the MFI of Cofilin-1 in the nucleus. The MFI of Cofilin-1 in the cytoplasm was quantified using ROIs defined by segmented lines within the whole cytoplasm. Image processing and analyses were performed using ImageJ (Fiji) software.

### 5-FUrd Incorporation

To quantify transcription, hUCM-MSCs were seeded at P2 and 48 h later were incubated with 2 mM of 5-fluorouridine (5-FUrd, Sigma-Aldrich) in proliferation medium during 15, 30, and 45 min at 37°C. Next, cells were washed once with cold PBS, permeabilised with 1% Triton X-100 for 20 min and fixed with 4% (w/v) PFA in PBS for another 20 min. 5-FUrd incorporation was analysed by ICC using an anti-BrdU antibody (Supplementary Table S2) previously described to recognise 5-FUrd (Obrdlik and Percipalle, 2011). Quantification of fluorescence levels was performed by calculating the corrected total cell fluorescence using DAPI to define ROIs and quantify the integrated density and nuclear area using Fiji software. The slopes were calculated by performing linear regression analysis of the corrected total cell fluorescence values of FUrd incorporation considering the time points between 15 and 45 min (GraphPad Prism 8).

### Cofilin-1 Gene Silencing

To knockdown Cofilin-1, hUCM-MSCs were transfected at P2 according to the manufacturer’s instructions on TCPS or custom-made 40:1 PDMS using lipofectamine 3000 (Thermo Fisher Scientific) with 150 nM of SignalSilence^®^ Cofilin siRNA I or SignalSilence^®^ Control siRNA as control (Cell Signalling Technology). 5-FUrd incorporation experiments were performed 72 h after transfection.

### Fluorescence Loss in Photobleaching (FLIP) and Image Analysis

Fluorescence Loss in Photobleaching (FLIP) was performed using a Zeiss LSM 710 confocal microscope with stage heated at 37.5°C and the Zen software was used for image acquisition (Zeiss). For this experiment MRC-5 cells were seeded at 15,000 cells/cm^2^ on 1.5 kPa PDMS or μ-slide well glass (both from IBIDI) and maintained in culture for 3 days. The experiment was performed according to Das Neves et al. (2010), Lima et al. (2018), and Steurer et al. (2018). Briefly, to photobleach GFP-RNA POL II in MRC-5 cells, rectangles covering approximately half of each nucleus were selected and 100% laser power was applied to bleach all fluorescent molecules in these areas. The bleaching acquisition cycles were run in a continuous mode during 1,800 s. A set of images were taken and the fluorescence intensity of GFP in the nucleus was quantified at the unbleached area using Fiji software. The fluorescence decay was plotted against the time of photobleaching, fitting data in a three-phase exponential decay curve to obtain half-life values attributed to the elongation time (GraphPad Prism 8).

### Statistical Analysis

Proteomics data were presented as the mean fold change of 40:1 PDMS over the respective TCPS sample for three biological replicates (umbilical cord samples from three different donors). Statistical analysis was performed using the SPSS^®^ Statistics V22 (IBM) for all proteins that presented PDMS/TCPS ratio with coefficient of variation (CV) below 30%. Data normality was assessed by Shapiro–Wilk test performed in infernoRDN and the One-Sample *t*-student test against a theoretical value of 1 was used to test the variations. Statistical significance was considered for *p* < 0.05.

Statistical analysis of the remaining data was performed using GraphPad Prism 8 software. Values are expressed as mean ± standard error of the mean (SEM) or median with range (as indicated) for at least three independent experiments. Differences between two groups were tested using Student’s *t*-test, One-Sample *t*-test (theoretical mean of 1) or the non-parametric Mann–Whitney test. Parametric analysis of variance (ANOVA) followed by Dunnett’s multiple comparisons test was used to compare more than two groups. For all statistical analysis, differences were considered significant for *p* < 0.05.

## Results

### Characterisation of Human Umbilical Cord Mesenchymal Stem/Stromal Cells (hUCM-MSCs) and 40:1 PDMS Substrates

**FIGURE 1:**
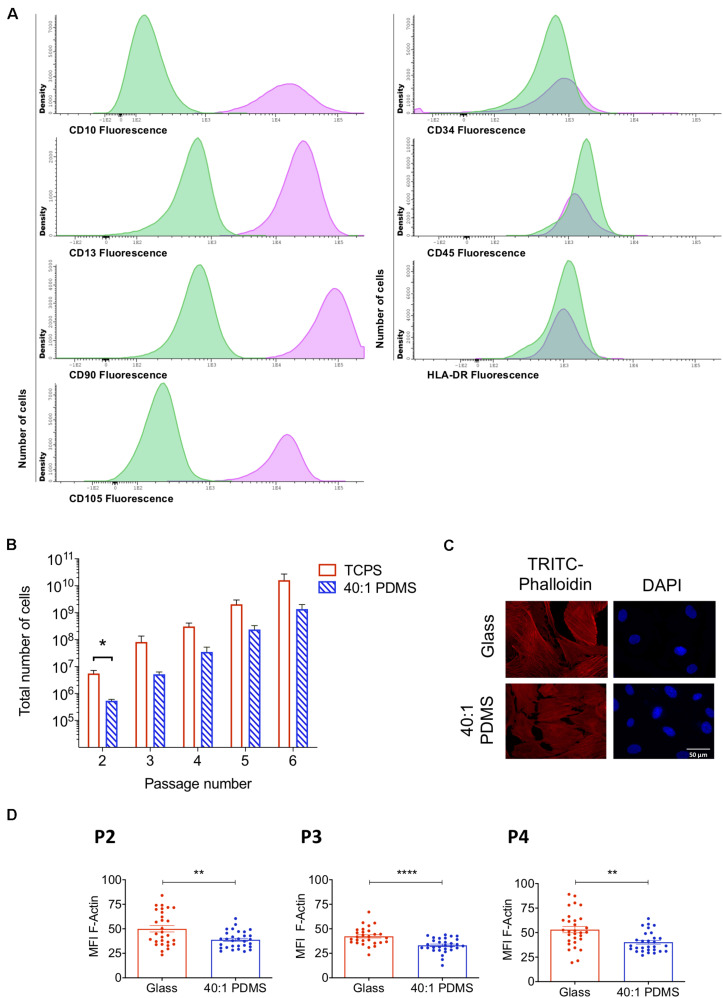
hUCM-MSCs can proliferate on and respond to mechanical cues provided by a soft PDMS substrate. **(A)** Immunophenotypic characterisation of hUCM-MSCs (average of four independent samples at P4). Cells were labelled with antibodies against the indicated antigens and analysed by flow cytometry. In parallel, unlabelled hUCM-MSCs were also acquired in the flow cytometer as negative controls. In the histograms, *y*-axis represents the number of cells (density); and *x*-axis represents the amount of protein expressed per cell, measured as mean fluorescence intensity (MFI). Labelled hUCM-MSCs are represented as pink lines, whereas green lines correspond to unlabelled hUCM-MSCs (negative control). hUCM-MSCs were positive for CD10, CD13, CD90, and CD105, and negative for CD34, CD45 and HLA-DR. **(B)** Total number of cells calculated for hUCM-MSCs on each passage (P2–P6) in culture on TCPS or 40:1 PDMS (as indicated). Bars represent mean ± SEM of at least three independent experiments using cells obtained from different donors. Statistical analysis was performed using the non-parametric Mann–Whitney test with significant differences indicated as *p < 0.05. **(C)** Representative fluorescence microscopy images and **(D)**, respective MFI of F-actin of hUCM-MSCs cultured on stiff or soft 40:1 PDMS substrates from P2 to P4 (as indicated). At each of the indicated passages, cells were seeded on stiff glass coverslips or soft 40:1 PDMS substrates for 48 h and then fixed and stained with TRITC-Phalloidin (to stain F-actin, in red) and DAPI (to counterstain nuclei, in blue). Bars represent mean ± SEM of three independent experiments. Statistical analysis was performed using a two-tailed Student’s *t*-test with significant differences indicated as **p < 0.01 and ****p < 0.0001.

MSCs are plastic-adherent cells that proliferate readily *in vitro* when maintained in standard culture conditions, being positive for CD105, CD73 and CD90 and negative for CD45, CD34, HLA-DR, CD14 or CD11B, CD79α or CD19 surface markers, as described by the International Society for Cellular Therapy (Dominici et al., 2006). For this study, we isolated hUCM-MSCs as previously described (Leite et al., 2014). The immunophenotypic characterisation of the cells in P4 by flow cytometry confirmed their identity, being positive for CD10, CD13, CD90, and CD105, and negative for CD34, CD45, and HLA-DR (Figure 1A).

For this study, polydimethylsiloxane substrates with a pre-polymer to curing agent ratio of 40:1 (40:1 PDMS) were produced for cell culture. The rheological analysis was performed to assess the shear elastic (storage modulus, *G*′) and viscous (loss modulus, *G*′′) properties of the substrates. A frequency sweep between 0.1 and 10 Hz was performed using a rheometer (Supplementary Figure S1B). Our results indicate that the elastic properties of the substrate are dominant, since *tan*⁡δ (*tan*⁡δ = *G*′′/*G*′) values were lower than 1 and *G*′ and *G*′′ were essentially independent of the measurement frequency (Rosales and Anseth, 2016) (Supplementary Figures S1B,C).

The Young’s modulus (*E*) was determined at 1 Hz as being 2870 ± 625 Pa (∼3 kPa) (Supplementary Figure S1C). To guarantee that the custom-made PDMS substrates reached the minimum thickness described to prevent cells from sensing the stiff supporting material underneath the elastomer (>100 μm) (Buxboim et al., 2010), substrate thickness was determined to be 315.5 ± 4.6 μm (Supplementary Figure S1), sufficient to allow the cells to sense only the soft material.

In order to evaluate the proliferative capacity of hUCM-MSCs when cultured on stiff (TCPS) or soft (40:1 PDMS) substrates, the total number of cells was determined between P2 and P6 (Figure 1B). In general, the total number of cells obtained on TCPS was higher when compared with PDMS, showing that cells cultured on soft substrates exhibit lower proliferative profile in comparison with those cultured on stiff TCPS (Figure 1B). Nevertheless, except in P2, no significant differences were found for the total number of cells, indicating some adaptation of the cells to the new soft environment. These results are in agreement with previous studies reporting higher cell proliferation on stiffer substrates (Provenzano and Keely, 2011). Additionally, population doubling, cumulative population doubling, and generation time were evaluated between P2 and P6 (Supplementary Figure S2), indicating a trend for slower proliferation kinetics of hUCM-MSCs on PDMS, but no significant differences were found.

To confirm that hUCM-MSCs respond to the distinct degrees of stiffness, P1 cells were seeded in parallel on glass coverslips (stiff) and 40:1 PDMS substrates (soft). After reaching P2, P3, or P4 on each substrate, F-actin levels were assessed to evaluate intracellular contractility, which is known to scale with the stiffness of the environment in mechanosensitive cells (Engler et al., 2006; Fu et al., 2010; Gerardo et al., 2019). As expected, F-actin levels were significantly lower in cells cultured on soft PDMS substrates when compared with cells maintained on stiff glass coverslips in all passages tested (Figures 1C,D).

### Substrate Stiffness Modulates the Proteome of hUCM-MSCs

**FIGURE 2:**
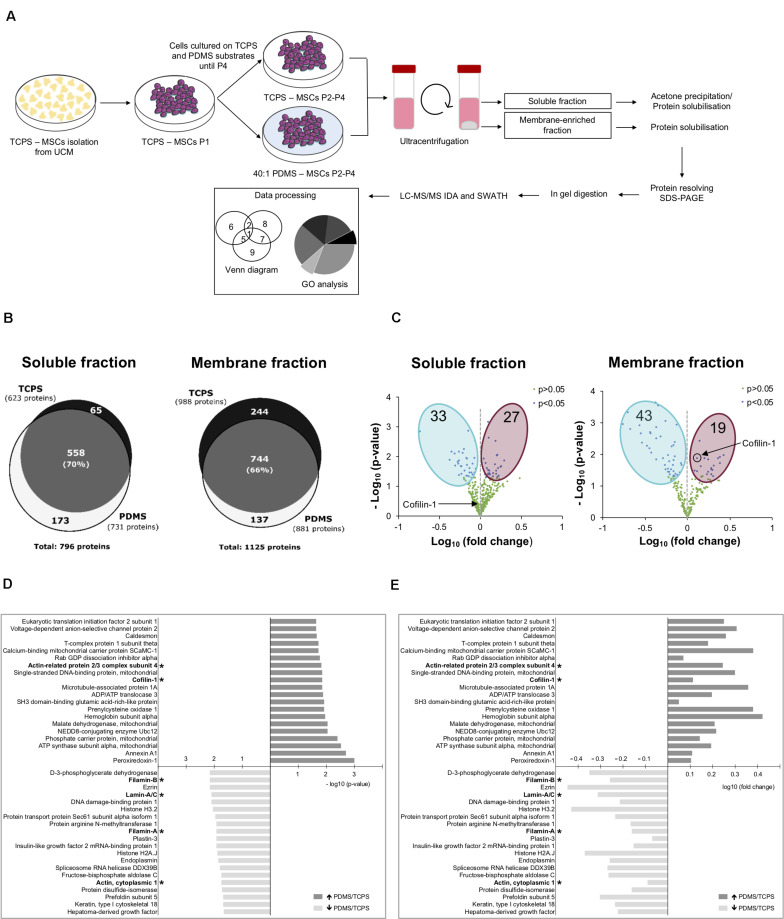
Substrate stiffness influences the proteome of hUCM-MSCs. **(A)** Experimental workflow. hUCM-MSCs were isolated from umbilical cord explants obtained from three distinct donors on TCPS plates and passaged (into P1) when the colonies were well developed. Cells were then maintained in parallel in culture from P2 until P4 on stiff TCPS and soft 40:1 PDMS substrates. Cells were lysed and then the membrane- and soluble-enriched fractions were obtained by ultra-centrifugation. The proteins from the two fractions were precipitated and/or solubilised, resolved by SDS-PAGE and analysed by mass spectrometry. **(B)** Venn diagrams illustrating the total number of proteins and the number of exclusive and common proteins identified in cells cultured on TCPS or 40:1 PDMS present in the soluble (left) or membrane (right) enriched fractions. **(C)** Volcano plots representing proteins with statistically significant differences (*p* < 0.05, blue dots) or non-significant differences (*p* > 0.05, green dots) between proteins found in the proteome of soluble (left) and membrane (right) fractions obtained from cells cultured on soft PDMS substrates relative to stiff TCPS. The blue and magenta areas surround the proteins with statistically significant lower or higher expression in cells cultured on soft PDMS versus stiff TCPS, respectively. Black circle and arrows pinpoint Cofilin-1 found in soluble (left) and membrane (right) fractions of cells cultured on soft PDMS versus stiff TCPS. Statistical analysis was performed using One-Sample *t*-test (theoretical mean of 1.0). **(D)** Bar chart representing the top 20 most significant (*p* < 0.05) and **(E)** differentially abundant proteins (fold change) present in soluble and membrane fractions obtained from cells cultured on 40:1 PDMS versus TCPS substrates. (*) marks proteins involved in mechanotransduction or actin cytoskeleton regulation. Bars represent –log10 of the *p*-value **(D)** or log10 of the fold change **(E)**. All data were collected from three independent experiments using cells obtained from three distinct donors.

To explore the influence of mechanical cues, and in particular the effect of substrate stiffness on the proteome of hUCM-MSCs, a SWATH-MS/MS proteomics analysis was performed using cells maintained on stiff TCPS or soft 40:1 PDMS substrates from P2 until P4 (workflow in Figure 2A). To obtain samples with less complexity (and to achieve a more comprehensive protein coverage), the intracellular contents were fractionated into soluble and membrane-enriched fractions. 796 proteins were identified in the soluble fraction, 558 of which were detected in cells cultured on both substrates (TCPS and 40:1 PDMS), representing 70% of the total number of proteins identified. On the other hand, 173 and 65 proteins were exclusively detected in the proteome of cells cultured on soft or stiff substrates (Figure 2B). Similarly, in the membrane-enriched fraction, 1125 proteins were identified, being 744 common for cells on TCPS and PDMS, representing 66% of the total proteins identified in that fraction. Hundred and thirty seven and 244 proteins were exclusively identified in the proteome of cells cultured on soft or stiff substrates (Figure 2B). To further characterize the differences between the cells cultured on the two systems, protein quantification was attempted to all the proteins identified (Figure 2C). Through SWATH-MS/MS analysis, 633 proteins were quantified in the soluble fraction obtained from cells cultured on both substrates, and among those 33 showed lower abundance, while 27 displayed higher expression in cells cultured in PDMS in comparison with TCPS (Figure 2C). In the membrane fraction, 624 proteins were quantified, with 43 and 19 proteins showing lower and higher expression, respectively, in cells maintained on the soft versus stiff substrate (Figure 2C).

Next, we analysed the twenty most statistically significant proteins more abundantly expressed in cells cultured on PDMS when compared with TCPS and vice versa (among both fractions). Data show that several of the identified proteins are involved in the regulation of actin cytoskeleton, hence putative modulators of mechanotransduction (Figures 2D,E). Within this category, Filamin-A/B, Lamin-A/C and Actin were more abundant in TCPS in comparison with PDMS, while subunit 4 of the Arp2/3 complex and Cofilin-1 were more expressed in cells in 40:1 PDMS when compared with TCPS (Figures 2D,E). Cofilin-1 presented good *p*-value and increased expression of approximately 1.3-fold (PDMS vs. TCPS; Supplementary Table S3) in the membrane fraction, being one of the most differentially expressed proteins in the proteome of hUCM-MSCs cultured on the soft when compared with the stiff substrate. Additionally, Cofilin-1 is a pivotal regulator of actin dynamics (Bamburg and Bernstein, 2010; Ohashi, 2015) and in turn the actin cytoskeleton is one the most important players in mechanotransduction (Harris et al., 2018). Hence, Cofilin-1 seemed to be a promising protein to focus on and to characterise further in the context of mechanotransduction and attempt to find implications in other important biological processes regulated by the protein such as transcription (Obrdlik and Percipalle, 2011; Percipalle, 2013).

### Cofilin-1 Is More Abundantly Present in Cells Cultured on Soft Versus Stiff Substrates

**FIGURE 3:**
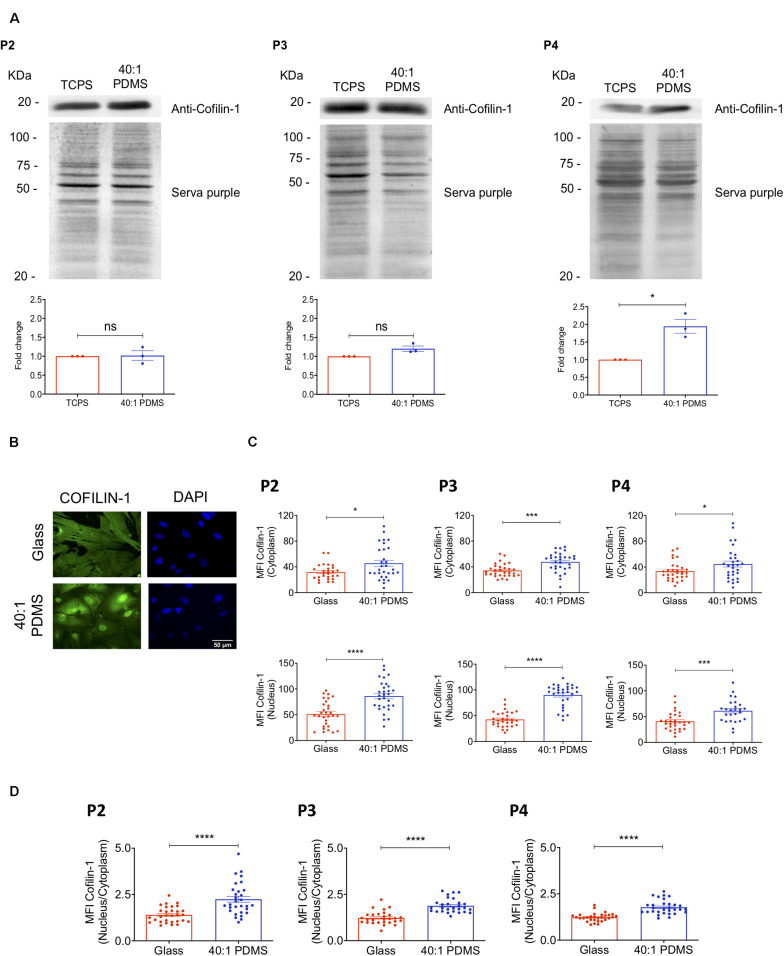
Cofilin-1 is highly present in cells cultured on soft versus stiff substrates. **(A)** Western blot analysis (top) of Cofilin-1 present in whole-cell protein extracts (separated by SDS-PAGE) obtained from hUCM-MSCs isolated and cultured on TCPS until P1 and then cultured on stiff TCPS or soft 40:1 PDMS until P2, P3, or P4 (as indicated). For quantification analysis (bottom), Cofilin-1 expression was normalised using the respective total protein level assessed by staining the WB membrane using SERVA purple (top). Bars (bottom) represent mean ± SEM of three independent experiments. Statistical analysis was performed using One-Sample *t*-test (theoretical mean of 1.0) with significant differences indicated as *p < 0.05; ns, non-significant. **(B)** Representative fluorescence microscopy images and **(C)** respective MFI quantification of Cofilin-1 in the nucleus or cytoplasm of cells cultured on glass coverslips or 40:1 PDMS until P2, P3, or P4 (as indicated). **(D)** MFI quantification of Cofilin-1 ratio (nucleus/cytoplasm) present in cells cultured as in **(C).** Cells were fixed and stained with an anti-Cofilin-1 antibody (green) and DAPI for nuclear counterstaining (blue). In **(C,D)** bars represent mean ± SEM of cells analysed from three independent experiments. Statistical analysis was performed using a two-tailed Student’s *t*-test, with significant differences indicated as *p < 0.05, ***p < 0.001, and ****p < 0.0001.

In order to validate and further explore the results obtained by mass spectrometry regarding Cofilin-1, western blot (WB) analysis was performed to measure the protein’s level in whole-cell extracts prepared from cells cultured on stiff (TCPS) or soft (40:1 PDMS) substrates until P2, P3, and P4. The results show that Cofilin-1 levels became gradually higher in cells cultured from P2 to P4 on the soft substrate when compared to those maintained on the stiffer one, achieving statistical significance in P4 (Figure 3A). This is consistent with the proteomics data regarding the increased presence of Cofilin-1 on the soft substrate in P4 (Figures 2D,E). As a control, we further analysed vinculin (Supplementary Figure S3), another protein related to mechanotransduction (Carisey et al., 2013) whose expression levels did not significantly change in the proteomics analysis (Supplementary Table S3). As expected, WB analysis confirmed the SWATH-MS/MS results.

To gain further insight into the regulation of the protein, the levels of Cofilin-1 were also assessed specifically in the nucleus and in the cytoplasm of cells cultured on soft and stiff (glass coverslips) substrates until P2, P3, and P4 by immunofluorescence microscopy. Data show a significant increase of the MFI of Cofilin-1 in both subcellular spaces (nucleus and cytoplasm) for all passages tested (Figures 3B,C). Taken together, these results strongly indicate that Cofilin-1 levels are higher in cells cultured on soft when compared with stiff substrates.

### Substrate Stiffness and Soluble Modulators of Actomyosin Influence Cofilin-1 Subcellular Localisation and Phosphorylation State

The immunofluorescence images of Cofilin-1 in hUCM-MSCs immediately suggested that the protein’s subcellular localisation might be influenced by substrate stiffness (Figure 3B). Image quantification revealed that the ratio of nuclear/cytoplasmic Cofilin-1 increased significantly in cells cultured on the soft substrates when compared with those on stiff glass coverslips (Figure 3D and Supplementary Figure S4). Cofilin-1 activity and subcellular localisation (Nebl et al., 1996) were reported to be largely regulated by phosphorylation on Serine 3 by its cognate kinase LIMK1 (Yang et al., 1998). Our results showed that after incubating cells with a pharmacological inhibitor of LIMK (LIMKi-3), the phosphorylation levels of Cofilin-1 on Ser3 drastically decreased in a dose-dependent manner (Supplementary Figure S5).

**FIGURE 4:**
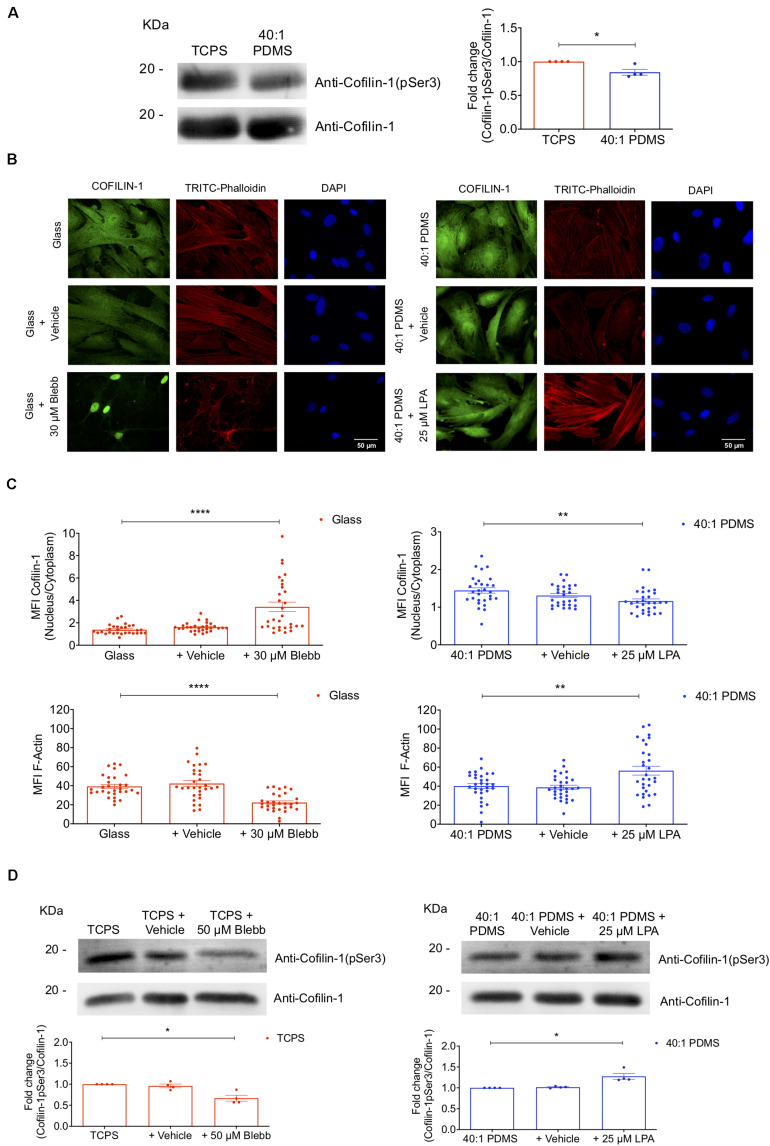
Substrate stiffness and soluble modulators of actomyosin influence Cofilin-1 subcellular localisation and phosphorylation state. **(A)** Cofilin-1 and phospho-Cofilin-1 (pSer3) levels were evaluated by western blot analysis of whole-cell protein extracts separated by SDS-PAGE (left) obtained from cells cultured on TCPS or 40:1 PDMS between P2 and P4. For the quantification (right) values were normalised by the respective total Cofilin-1 protein level in TCPS or PDMS for each independent experiment. Bars represent the mean of the ratio of Cofilin-1 (pSer3)/total Cofilin-1 ± SEM of four independent experiments. Statistical analysis was performed using One-Sample *t*-test (theoretical mean of 1.0) with significant differences indicated as *p < 0.05. **(B)** Representative fluorescence microscopy images and **(C)** respective MFI quantification of Cofilin-1 nucleus/cytoplasm ratio and F-actin (TRITC-Phalloidin) for cells cultured on glass coverslips or 40:1 PDMS after treatment or not with Blebbistatin or LPA (as indicated). Cells seeded on stiff glass coverslips (left) were cultured for 24 h and then incubated or not with Blebbistatin (30 μM) for an additional 24 h; cells seeded on soft 40:1 PDMS (right) were cultured for 46 h and then incubated or not with LPA (25 μM) for an additional 2 h. In both cases, cells were fixed after 48 h in culture and stained with anti-Cofilin-1 antibody (green), TRITC-Phalloidin for F-actin (red) and DAPI for nuclear counterstaining (blue). Bars represent mean ± SEM of cells analysed from three independent experiments. Statistical analysis was performed using One-Way ANOVA followed by Dunnett’s multiple comparisons test between all conditions (**p < 0.01; ****p < 0.0001). **(D)** Western blot analyses were performed to detect Cofilin-1 and phospho-Cofilin-1 (pSer3) as described in **(A)**, using whole-cell extracts obtained from cells cultured on stiff TCPS and treated or not with Blebbistatin or cultured on soft 40:1 PDMS and treated or not with LPA (as indicated), with seeding and treatment regimens similar to those described in **(B,C)**. For the quantification (bottom) values were normalised by the respective total Cofilin-1 protein level in TCPS (left) or PDMS (right) for each independent experiment. Bars represent the mean of the ratio of Cofilin-1 (pSer3)/total Cofilin-1 ± SEM of four independent experiments. Statistical analysis was performed using One-Sample *t*-test (theoretical mean of 1.0) with significant differences indicated as *p < 0.05.

It is also described that phospho-Cofilin-1 (p-Cofilin-1) is inactive and remains in the cytosol. When dephosphorylated, Cofilin-1 is active and hence able to severe and depolymerise actin filaments into globular actin, subsequently remaining bound to G-actin (Percipalle, 2013). Then, still bound to G-actin, Cofilin-1 translocates into the nucleus through a mechanism mediated by importin 9 (Dopie et al., 2012; Percipalle, 2013). To assess if the changes in subcellular localisation of Cofilin-1 in response to substrate stiffness correlated with the phosphorylation state of the protein, we measured the levels of *p*-Cofilin-1 on Ser3 and total Cofilin-1 (by western blot analysis) in cells cultured on TCPS and 40:1 PDMS. We observed a significant decrease in the ratio of Cofilin-1 (pSer3)/total Cofilin-1 (Figure 4A) in cells cultured on the soft in comparison with those on the stiff substrate. The phosphorylation of Cofilin-1 on Ser3 was also detected by proteomics analysis. The ratio of Cofilin-1 (pSer3)/total Cofilin-1 measured in cells cultured on soft (PDMS) was only about 40% of the value found in cells maintained on stiff (TCPS) until P4 (Supplementary Figure S6). Hence, data indicate that the expected correlation between the subcellular localisation and phosphorylation state of Cofilin-1 reported in the abovementioned literature holds true when these changes occur in response to substrate stiffness.

To further understand the response of Cofilin-1 to mechanical stimuli in terms of subcellular localisation and phosphorylation state, we devised an experiment to mimic stiff conditions while cells were cultured on a soft substrate and vice versa. To that end, cells were seeded on stiff glass coverslips or soft 40:1 PDMS substrates and then incubated with Blebbistatin or LPA, respectively. Blebbistatin is a NMM-II inhibitor, causing relaxation of the cellular actin network (typical of cells cultured on a soft condition) (Engler et al., 2006; Lourenco et al., 2016) and LPA leads to activation of RhoA, inducing actomyosin contractility (typical of cells on stiff substrates) (Sun et al., 2014), hence mimicking a soft condition on stiff substrates and vice versa. When cells on glass coverslips were incubated with Blebbistatin, the ratio of nuclear/cytosolic Cofilin-1 increased significantly compared to untreated cells. Conversely, cells incubated with LPA cultured on soft PDMS showed a significant decrease in the nuclear/cytosolic ratio of the protein when compared to the respective control (Figures 4B,C). Additionally, we analysed the levels of F-actin upon incubation with Blebbistatin or LPA using TRITC-phalloidin. As expected, a significant decrease or increase of F-actin was observed in cells incubated with Blebbistatin or with LPA (respectively) comparing with their respective controls (Figures 4B,C), indicating that the treatments with the actomyosin-modulating soluble-factors tested were indeed effective.

To assess the phosphorylation state of Cofilin-1 in cells incubated with Blebbistatin or LPA, WB analysis was performed. After incubation with Blebbistatin, the ratio of Cofilin-1(pSer3)/total Cofilin-1 in cells cultured on a stiff substrate decreased significantly. Conversely, in cells incubated with LPA while cultured on a soft substrate, this ratio was significantly higher than in control cells (Figure 4D).

These results indicate that cells cultured on stiff substrates (or on soft but in the presence of LPA) display high intracellular actomyosin tension (Figures 1C, 4B), which is accompanied by a low nuclear/cytosolic ratio distribution of Cofilin-1 and high phosphorylation of the protein on Ser3 (Figures 4A,D). Conversely, MSCs cultured on soft substrates (or on stiff but in the presence of Blebbistatin) present low intracellular contractility (Figures 1C, 4B), which correlates with a high ratio of nuclear/cytosolic distribution of Cofilin-1 and low phosphorylation of the protein on Ser3 (Figures 4A,D).

### hUCM-MSCs Respond to Soft Substrates With Increased Overall Transcription in a Cofilin-1-Dependent Manner

**FIGURE 5:**
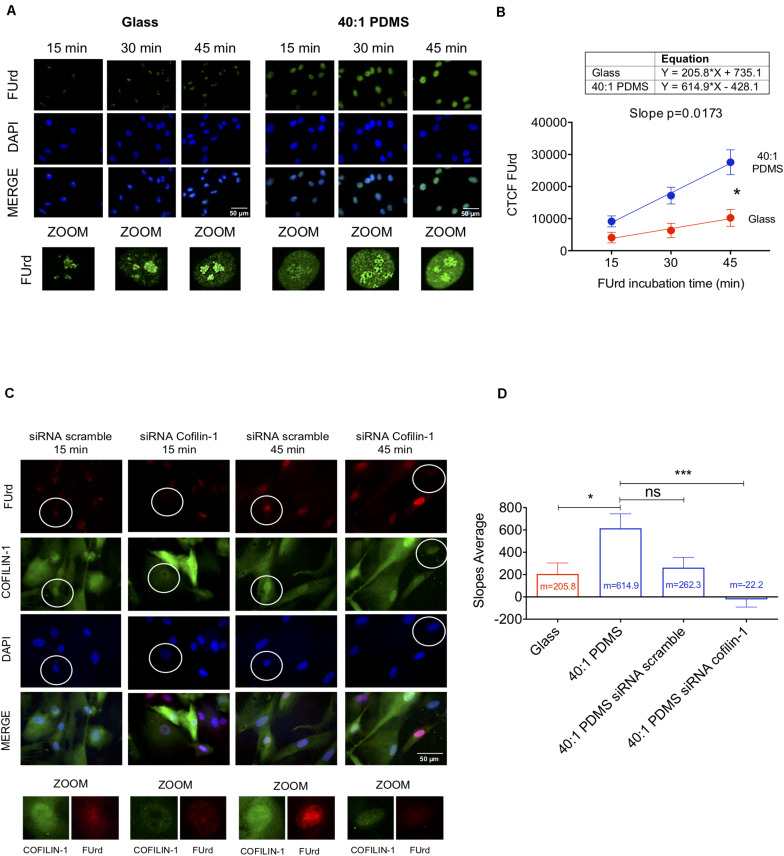
Soft substrates induce overall increased transcription in hUCM-MSCs in a Cofilin-1-dependent manner. **(A)** Representative fluorescence microscopy images of the nuclei of cells cultured on glass coverslips or 40:1 PDMS. After 48 h in culture, hUCM-MSCs were incubated with FUrd during 15, 30, and 45 min, fixed and stained with an anti-BrdU antibody that recognises FUrd (green) to identify the new transcripts and nuclei were counterstained with DAPI (blue). **(B)** Linear regression of FUrd nuclear incorporation (CTCF, corrected total cell fluorescence) as a function of time occurring in cells on each substrate (data represent mean ± SEM of 6 independent experiments). Linear regression analysis (using the linear regression tools of GraphPad Prism 8) shows that the slopes of the two curves are significantly different from each other (*p* = 0.0173), indicating increased transcriptional activity in cells cultured on soft PDMS substrates (blue line) when compared with glass (red). **(C)** Representative fluorescence microscopy images of FUrd incorporation during 15 and 45 min in control and Cofilin-1 knock-down cells (using siRNA). Cells were immunostained with anti-Cofilin-1 (green) and anti-BrdU/FUrd (red) antibodies and nuclei were counterstained with DAPI (blue). **(D)** Bars represent the mean ± SEM of the slope values of FUrd incorporation (as determined in **B**) for each of the indicated conditions. Only cells effectively knocked-down for Cofilin-1 (representative images highlighted with circles) were taken into account during corrected total cell fluorescence quantification of FUrd. Statistical analysis was performed for 6 independent experiments using One-Way ANOVA followed by Dunnett’s multiple comparisons test comparing all conditions against 40:1 PDMS (ns, non-significant; *p < 0.05; ***p < 0.001).

Knowing that Cofilin-1 (in combination with G-actin) is required for RNA polymerase II-mediated transcription elongation (Obrdlik and Percipalle, 2011; Percipalle, 2013), we sought to elucidate if the increased presence of Cofilin-1 in the nucleus of hUCM-MSCs cultured on soft substrates correlated with increased transcription. Hence, to measure global transcriptional activity, cells (cultured on stiff glass coverslips or soft 40:1 PDMS) were subjected to a FUrd pulse-chase (Obrdlik and Percipalle, 2011). We observed that FUrd incorporation in cells maintained on stiff substrates occurred mostly in discrete nucleolar *foci* for short incubation times, with increasing nucleoplasmic signal with time (Figure 5A), as validated by ICC analysis against the nucleolus-associated protein Nucleolin (Supplementary Figure S7). On the other hand, for cells cultured on the 40:1 PDMS substrate, data show multiple and more intense nucleoplasmic *foci* (Figure 5A) in addition to the nucleolar signal similar to that observed on the stiff condition. This strongly suggests that the increase in FUrd incorporation observed in cells on the soft substrate relies on RNA polymerase II- (with nucleoplasmic localisation) and not on RNA polymerase I- (with nucleolar localisation) mediated transcription (Szentirmay and Sawadogo, 2000). Quantification of FUrd incorporation over time showed that the slope of the curve within a linear range (between 15 and 45 min of incorporation) was significantly higher in cells cultured on the soft substrate when compared to the stiff (Figure 5B). Since the slope is proportional to the overall transcription, these results indicate that soft 40:1 PDMS substrates enhance hUCM-MSCs transcriptional activity by favouring the formation of nascent transcripts.

To assess if the increase of transcriptional activity in cells cultured on soft substrates is Cofilin-1-dependent, we performed similar FUrd pulse-chase experiments after performing Cofilin-1 knockdown using siRNA (as confirmed by western blot and fluorescence microscopy in Supplementary Figure S8). Data show that in cells in which Cofilin-1 was effectively silenced, the transcriptional activity was drastically decreased, resulting in few to almost non-existent nucleoplasmic *foci* (Figure 5C). Quantification analysis of the FUrd incorporation curve slopes revealed that the transcriptional activity in untreated cells cultured on soft substrates (40:1 PDMS) was not significantly different from those treated with scrambled siRNA, but was statistically higher than in hUCM-MSCs in which Cofilin-1 was effectively silenced (siRNA Cofilin-1), as well as in cells left untreated but maintained on a stiff substrate (glass), which were used as a control (Figure 5D). Hence, data indicate that soft substrate-induced increase of transcriptional activity observed in hUCM-MSCs is, at least in part, dependent on Cofilin-1.

### RNA Polymerase II Transcription Elongation Is Faster in Cells Cultured on Soft PDMS Substrates

**FIGURE 6:**
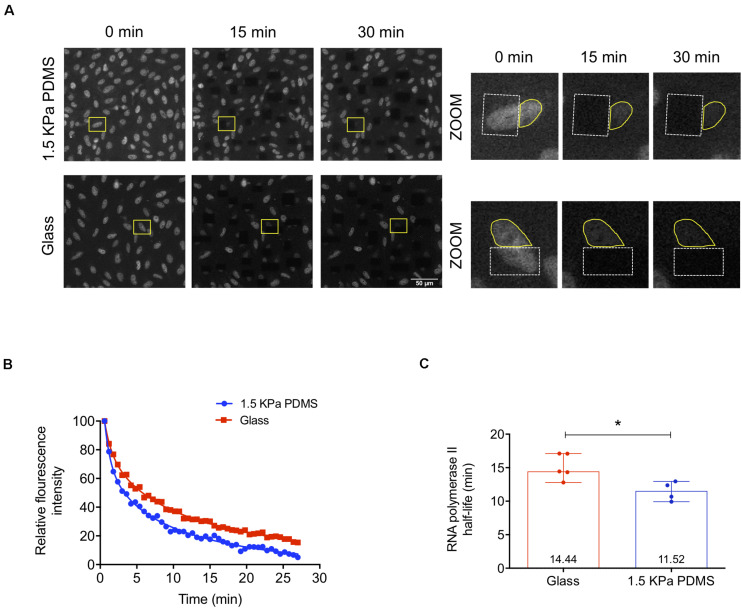
RNA polymerase II transcription elongation is faster in cells cultured on soft PDMS substrates. **(A)** Representative confocal microscopy images of MRC-5 cells (expressing GFP-RNA polymerase II) during fluorescence loss in photobleaching (FLIP) experiments. MRC-5 cells were cultured on stiff μ-slide glass wells or soft 1.5 kPa PDMS and after 72 h in culture cells were subjected to FLIP assay. In the zoomed images on the right panel (from the highlighted regions indicated with the yellow rectangles on the left panel), the dashed white lines circumscribe the bleached areas and the solid yellow lines represent the fluorescence measurement areas. **(B)** Representative three-phase exponential decay curves of GFP-RNA polymerase II signal of two single cells cultured on each substrate (glass or PDMS). **(C)** RNA polymerase II elongation half-life time was calculated based on the half-time slow parameter of the three-phase exponential decay curves obtained from cells cultured on stiff or soft substrates. Bars represent the median with range of at least four independent experiments. For each experiment, at least 10 cells were quantified. Statistical analysis was performed using the non-parametric Mann–Whitney test, with significant differences indicated as *p < 0.05.

To understand if the enhanced transcriptional activity measured in cells cultured on soft versus stiff substrates was at least in part due to faster RNA polymerase II transcription elongation, the mechanism in which Cofilin-1 is described to be involved in (reviewed by Percipalle, 2013), we performed FLIP assays. To that end, we used human fibroblasts engineered with GFP-RNA polymerase II (MRC-5 cells) (Steurer et al., 2018) cultured for 3 days on stiff glass or soft 1.5 kPa PDMS. This was the time required to verify Cofilin-1 nuclear distribution (Supplementary Figure S9A) similar to that observed in hUCM-MSCs on a soft substrate, hence demonstrating that MRC-5 cells were mechanoresponsive. Additionally, similar to hUCM-MSCs, Cofilin-1 in MRC-5 cells also responded to soluble modulators of actomyosin contractility (Supplementary Figure S9B), with a significant increase or decrease of the ratio of nuclear/cytosolic Cofilin-1 in cells treated with Blebbistatin or LPA (respectively). Regarding FLIP, data show a faster decay of fluorescence in cells cultured on soft PDMS when compared with those cultured on stiff conditions, indicating that the transcriptional elongation is faster on soft substrates (Figure 6A). By quantifying the fluorescence decay in single cells as a function of time during the FLIP assay allowed the creation of three-phase exponential decay curves (Figure 6B). By analysing curves parameters attributed to the elongation phase — half-life slow (Das Neves et al., 2010; Lima et al., 2018; Steurer et al., 2018) —, the elongation half-life time of RNA polymerase II in cells cultured on the soft substrate was significantly lower than that in cells maintained on stiff conditions (Figure 6C). This indicates that RNA polymerase II transcription elongation speed is higher in cells maintained on soft than on stiff substrates.

The overall results indicate that the increased presence of Cofilin-1 in the nucleus of mechanosensitive cells in response to soft substrates facilitates transcription, through a mechanism that is consistent with enhanced RNA polymerase II transcription elongation speed.

## Discussion

This study explores the effects of substrate stiffness in the proteome of proliferating undifferentiated hUCM-MSCs. *In vitro* expansion of MSCs has been associated with the loss of cell potency due to extensive proliferation, which is required for example, to obtain a clinically relevant number of cells for therapeutic purposes (reviewed in Hoch and Leach, 2014; Müller et al., 2015; Galipeau et al., 2016). Recently, there is growing evidence suggesting that such loss of potency may at least in part be related to the mechanical properties of standard cell-culture substrates (Lee et al., 2014; Yang et al., 2014; Kusuma et al., 2017), like TCPS, much stiffer than most tissues present in biological systems.

MSCs are well known for being highly mechanosensitive cells, scaling intracellular contractility according to the stiffness of the surrounding environment (Fu et al., 2010; Swift et al., 2013; Gerardo et al., 2019), which greatly affects their differentiation potential toward distinct lineages (Engler et al., 2006; Fu et al., 2010; Gao et al., 2010), as well as reprogramming into iPSCs (as previously shown by our laboratory (Gerardo et al., 2019). However, the impact of mechanical cues and in particular of substrate stiffness in proliferating undifferentiated MSCs is still largely unknown.

Our results demonstrate that the proteome of hUCM-MSCs presents differences between cells cultured (at least between P2 and P4) on stiff TCPS (standard cell culture conditions) or on soft PDMS substrates. A putative limitation is that the TCPS used for the proteomics approach was not coated with the ECM proteins used on the PDMS substrate, although the glass coverslips used for subsequent experiments were treated in a way similar to the elastomeric surface. In this study, we focussed our attention on proteins that could be identified in both cell culture conditions, but whose relative abundance was significantly different. Among such proteins, many are involved in the regulation and modulation of the actin cytoskeleton, hence being putative good candidates to be involved in mechanotransduction mechanisms. Within this group, Filamin-A/B, Lamin-A/C and Actin were found to be more abundantly present in TCPS in comparison with PDMS. To our knowledge, Filamin-A/B and Actin were not previously reported to change their levels in response to changes in substrate stiffness. Concerning Lamin-A, our data is in line with previous studies showing that the protein is mechanoresponsive and that its levels scale with the stiffness of the microenvironment (Swift et al., 2013; Swift and Discher, 2014; Toh et al., 2015). Similar to subunit 4 of the Arp2/3 complex (involved in the regulation of actin cytoskeleton (May, 2001), Cofilin-1 was one of the proteins whose levels increased mostly in the proteome of hUCM-MSCs cultured on soft PDMS compared to stiff TCPS substrates (as confirmed by western blot and ICC). For that reason and being Cofilin-1 an important actin-regulating protein, together with the lack of information about its role in the context of cellular mechanobiology, our studies became focussed on this protein.

ICC analysis revealed that Cofilin-1 present in hUCM-MSCS cultured on soft substrates exhibited a preferential nuclear localisation, in contrast with cells on stiff substrates, in which Cofilin-1 presented a more widespread distribution. Moreover, the phosphorylation state of Cofilin-1 [by its cognate kinase LIMK1 (Yang et al., 1998)] in hUCM-MSCs cultured in soft versus stiff substrates also changed. WB analysis showed that the ratio of Cofilin-1 (pSer3)/total Cofilin-1 in hUCM-MSCs cultured on soft substrates was significantly lower than when cultured on stiff conditions (which was also supported by the proteomics data). Our results are in line with a report showing that mechanical force applied to cells via integrins caused Rho/ROCK/LIMK-dependent phosphorylation of Cofilin-1 (Zhao et al., 2007). Moreover, as it was previously shown using Cofilin-1 Ser3 phosphorylation mutants, the non-phosphorylated mimetic (Ser3→Ala) tends to accumulate in the nucleus, while the WT is consistent with a predominantly cytosolic localisation (Nebl et al., 1996). Taken together, our results strongly indicate that Cofilin-1 nuclear localisation increases when cells are cultured on soft substrates, and in these conditions Cofilin-1 is less phosphorylated. On the other hand, on stiff substrates, the protein presents a widespread localisation and is highly phosphorylated on Ser3 by LIMK1. Additionally, Cofilin-1 and other cytoskeletal proteins were recently identified as novel neddylation substrates, suggesting that this post-translational modification could generally modulate cytoskeletal proteins (Vogl et al., 2020). Specifically, Cofilin-1 activity seems to increase when neddylation is inhibited, at least in neurons. Interestingly, NEDD8-conjugating enzyme Ubc12, one of the pivotal proteins for neddylation, was found more abundantly present in the proteome of hUCM-MSCs cultured on the soft 40:1 PDMS substrate (as shown in the proteomics data). This observation prompts us to speculate that regulation of the cytoskeleton by mechanotransduction may involve neddylation of cytoskeletal proteins, and Cofilin-1 in particular, which should be explored in future studies.

The Cofilin-1 localisation data is also consistent with literature indicating that Cofilin-1 is not able to bind to actin filaments which are under tension and/or populated by myosin (Ngo et al., 2016), which would be expected to occur in cells cultured on stiff substrates. We can speculate that, if Cofilin-1 is not able to bind to and depolymerise F-actin filaments into G-actin, the protein should not be able to migrate to the nucleus, since both Cofilin-1 and G-actin seem to migrate together with importin-9 (Pendleton et al., 2003; Dopie et al., 2012).

Knowing that Blebbistatin inhibits NMM-II, promoting the relaxation of the actin cytoskeleton (Engler et al., 2006; Lourenco et al., 2016), and that LPA induces intracellular contractility by activating RhoA and consequently favouring NMM-II activity (Sun et al., 2014), we sought to promote changes in the intracellular tension of hUCM-MSCs to further study the role of Cofilin-1 in mechanotransduction. Hence, by mimicking soft conditions on stiff substrates, and vice versa, using an inhibitor or a stimulator of actomyosin contractility (Blebbistatin or LPA, respectively), we confirmed that hUCM-MSCs respond to these modulators with a significant increase of the nuclear/cytosolic ratio of Cofilin-1 in response to Blebbistatin and a significant decrease in cells incubated with LPA. Moreover, the phosphorylation of Cofilin-1 on Ser3 decreased upon incubation with Blebbistatin and increased in response to LPA. Hence, Cofilin-1 seems to change in terms of intracellular localisation and phosphorylation state in response to low (Blebbistatin or soft substrates) or high (LPA or stiff matrices) intracellular contractility, reinforcing its role as a mechanotransduction player.

It has been reported that Cofilin-1 dephosphorylated on Ser3 is the active form of the protein, becoming capable to severe and depolymerise F-actin (Wioland et al., 2017). As a consequence of F-actin severing or depolymerisation activity, Cofilin-1 remains bound to G-actin, forming a complex that is imported into the nucleus (Dopie et al., 2012; Percipalle, 2013). Hence, our results regarding the increased nuclear presence of Cofilin-1 and its lower phosphorylation on Ser3 (in cells cultured on soft substrates or treated with Blebbistatin) are consistent with the abovementioned literature.

Moreover, our results indicate that soft substrates induce an increase of hUCM-MSCs overall transcriptional activity. In the nucleus, Cofilin-1 (in association with G-actin) was reported to be required for RNA polymerase II transcription elongation (Obrdlik and Percipalle, 2011; Percipalle, 2013). In fact, as a consequence of Cofilin-1 gene silencing, we observed a global decrease of transcription in cells cultured on soft substrates with few to almost non-existent nucleoplasmic foci (which are associated with RNA polymerase II activity) (Szentirmay and Sawadogo, 2000). By performing FLIP assays, we observed that the half-life elongation time of RNA polymerase II was significantly lower in mechanoresponsive fibroblasts (MRC-5 cells expressing GFP-RNA polymerase II) cultured on soft when compared with those cultured on a stiff substrate. This result indicates a higher RNA polymerase II transcription elongation speed in cells cultured on soft comparing to stiff substrates. Therefore, our findings strongly suggest that in soft conditions Cofilin-1 localises preferentially in the nucleus, facilitating transcription through the enhancement of RNA polymerase II transcription elongation.

Overall, our data indicate that Cofilin-1 is a central player of a newly identified mechanism coupling mechanotransduction and regulation of transcription, opening a new avenue for future studies in this field. Other mechanisms involved in the regulation of transcription in response to mechanotransduction stimuli have been reported. Some involve mechanosensitive transcription factors (Mammoto et al., 2012), like YAP/TAZ (Dupont et al., 2011; Morgan et al., 2013), Runx2 (Kanno et al., 2007) or NKX2.5 (Dingal et al., 2015). In fact, the LIMK/Cofilin-1 pathway was shown to modulate the activity of YAP/TAZ by regulating the formation of F-actin and stress fibres. Cofilin-1 depletion leads to high F-actin content and consequent increase in YAP/TAZ nuclear localisation, transcriptional activity, and cell proliferation (Aragona et al., 2013). Other involve changes in nuclear architecture and modulation of chromosome territories. The existence of physical links and bi-directional force transmission between the ECM and the nucleus (namely through the protein-protein interactions existing between integrins and focal adhesion proteins, the cytoskeleton, the LINC complex and the nucleoskeleton) (Jaalouk and Lammerding, 2009; Wang et al., 2009; Yu et al., 2009) influence the nuclear architecture and in turn modulate the genome’s organisation and gene expression (Wang et al., 2009; Shivashankar, 2011, 2019). Another example is a group of epigenetic changes occurring in response to mechanical cues, which can be generically encompassed within the concept of mechanoepigenetics (Missirlis, 2016). Some of the best-known mechanisms concerning chromatin epigenetic modifications in response to mechanical stimuli involve changes in histone acetylation and/or methylation (Downing et al., 2013; Hernandez et al., 2016; Roy et al., 2018; Gerardo et al., 2019), although many of the detailed mechanisms involved remain elusive.

**FIGURE 7:**
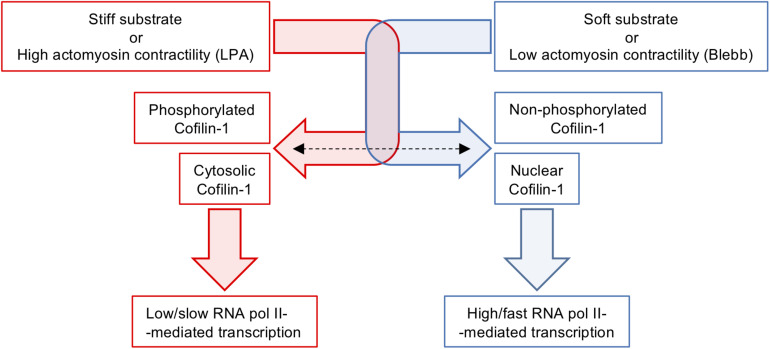
Schematics illustrating the modulation of Cofilin-1 by substrates stiffness or soluble factors affecting intracellular contractility. Stiff substrates or actomyosin tension induced by LPA favour elevated levels of F-actin, Cofilin-1 phosphorylation and Cofilin-1 adopts a mainly cytosolic localisation. On the other hand, soft substrates or low actomyosin tension caused by treatment with Blebbistatin induce low amounts of F-actin, no Cofilin-1 phosphorylation and Cofilin-1 adopts a nuclear localisation. The presence of Cofilin-1 in the nucleus is consistent with enhanced/faster RNA polymerase II-dependent transcription.

We showed that the proteome of proliferating and undifferentiated hUCM-MSCs changes depending on the stiffness of the extracellular environment. As a consequence of such finding, we identified and characterised Cofilin-1 as a new mechanotransduction player that responds (in terms of abundance, intracellular localisation and phosphorylation/activation state) to changes in the extracellular stiffness and intracellular contractility, playing a significant role in transcription mediated by RNA polymerase II (Figure 7). This study contributes with new fundamental knowledge in cell biology and in particular in the field of cellular mechanobiology, further establishing hUCM-MSCs as being highly mechanosensitive. It also shows that substrate stiffness is a highly relevant aspect for the expansion of this cell type *in vitro*, with impact in both research and clinical settings.

## Data Availability Statement

The datasets presented in this study can be found in online repositories. The names of the repository/repositories and accession number(s) can be found below: http://www.proteomexchange.org/, PXD017674.

## Ethics Statement

The studies involving human participants were reviewed and approved by the Ethics Committee of the Faculty of Medicine, University of Coimbra, Portugal (ref. CE-075/2019). Written informed consent to participate in this study was provided by the participants’ legal guardian/next of kin.

## Author Contributions

CD contributed to the design of the project, performed most of the experiments and data analysis, and prepared the manuscript. AG performed the MSC proliferation experiments and mass spectrometry experiments. SA performed the mass spectrometry experiments, proteomics data analysis and interpretation. AM and CA performed the FLIP experiments. IC and JL-S performed the rheological characterisation of PDMS substrates. JL-S contributed with the interpretation of the data. AP performed the immunophenotypic characterisation of hUCM-MSCs and data interpretation. JC contributed with the design and interpretation of the data. RP designed and performed the FLIP experiments and contributed to transcription-related data analysis and interpretation. BM contributed with the interpretation of the mass spectrometry proteomics data and with financial support. MG conceived and designed the project, interpreted the data, prepared the manuscript, and contributed with financial support. All the authors read and approved the final manuscript.

## Conflict of Interest

The authors declare that the research was conducted in the absence of any commercial or financial relationships that could be construed as a potential conflict of interest.

## Abbreviations

5-FUrd: 5-fluorouridine
COL-1: Type-I collagen
CV: coefficient of variation
DMSO: dimethyl sulfoxide
*E*: Young’s modulus
ECF: enhanced chemifluorescence
ECM: extracellular matrix
F-actin: filamentous actin
FAK: focal adhesion kinase
FAs: focal adhesions
FBS: foetal bovine serum
FLIP: fluorescence loss in photobleaching
FN: fibronectin
G-actin: globular actin
**G*′*: Elastic/Storage modulus
*G*′′: Viscous/Loss modulus
hUCM-MSCs: human umbilical cord matrix mesenchymal stem/stromal cells
ICC: immunocytochemistry
IDA: information-dependent acquisition
LIMK1: LIM Kinase 1
Limki-3: LIM Kinase inhibitor
LINC: Linker of nucleoskeleton and cytoskeleton
LPA: lysophosphatidic acid
mAbs: monoclonal antibodies
MFI: mean of fluorescence intensity
NMM-II: non-muscle myosin-II
ON: overnight
PDMS: polydimethylsiloxane
PFA: paraformaldehyde
PVDF: polyvinylidene fluoride
ROCK: rho-associated protein kinase
ROIs: regions of interest
RT: room temperature
SEM: standard error of the mean
SWATH-MS: sequential window acquisition of all theoretical fragment-ion spectra mass spectrometry
TCPS: tissue culture polystyrene
UCM: umbilical cord matrix
WB: western blot.

## References

[B1] Anjo S. I. Santa C. Manadas B. (2015). Short GeLC-SWATH: a fast and reliable quantitative approach for proteomic screenings. *Proteomics* 15 757–762. 10.1002/pmic.201400221 25418953

[B2] Anjo S. I. Santa C. Saraiva S. C. Freitas K. Barah F. Carreira B. (2017). “Neuroproteomics using short GeLC-SWATH: from the evaluation of proteome changes to the clarification of protein function,” in *Current Proteomic Approaches Applied to Brain Function*, eds Santamaría E. Fernández-Irigoyen J. (Cham: Springer), 107–138. 10.1007/978-1-4939-7119-0_8

[B3] Aragona M. Panciera T. Manfrin A. Giulitti S. Michielin F. Elvassore N. (2013). A mechanical checkpoint controls multicellular growth through YAP/TAZ regulation by actin-processing factors. *Cell* 154 1047–1059. 10.1016/j.cell.2013.07.042 23954413

[B4] Bamburg J. R. Bernstein B. W. (2010). Roles of ADF/cofilin in actin polymerization and beyond. *F1000 Biol. Rep*. 2:62. 10.3410/B2-62 PMC2990448 21173851

[B5] Burridge K. Guilluy C. (2016). Focal adhesions, stress fibers and mechanical tension. *Exp. Cell Res.* 343 14–20. 10.1016/j.yexcr.2015.10.029 26519907 PMC4891215

[B6] Burridge K. Monaghan-Benson E. Graham D. M. (2019). Mechanotransduction: from the cell surface to the nucleus via RhoA. *Philos. Trans. R Soc. Lond. B Biol. Sci.* 374:20180229. 10.1098/rstb.2018.0229 31431179 PMC6627015

[B7] Buxboim A. Rajagopal K. Brown A. E. Discher D. E. (2010). How deeply cells feel: methods for thin gels. *J. Phys. Condens. Matter.* 22:194116 10.1088/0953-8984/22/19/194116 PMC2864502 20454525

[B8] Carisey A. Tsang R. Greiner A. M. Nijenhuis N. Heath N. Nazgiewicz A. (2013). Vinculin regulates the recruitment and release of core focal adhesion proteins in a force-dependent manner. *Curr. Biol.* 23 271–281. 10.1016/j.cub.2013.01.009 23375895 PMC3580286

[B9] Collins B. C. Gillet L. C. Rosenberger G. Rost H. L. Vichalkovski A. Gstaiger M. (2013). Quantifying protein interaction dynamics by SWATH mass spectrometry: application to the 14-3-3 system. *Nat. Methods* 10 1246–1253. 10.1038/nmeth.2703 24162925

[B10] Das Neves R. P. Jones N. S. Andreu L. Gupta R. Enver T. Iborra F. J. (2010). Connecting variability in global transcription rate to mitochondrial variability. *PLoS Biol.* 8:e1000560. 10.1371/journal.pbio.1000560 21179497 PMC3001896

[B11] Dingal P. C. Bradshaw A. M. Cho S. Raab M. Buxboim A. Swift J. (2015). Fractal heterogeneity in minimal matrix models of scars modulates stiff-niche stem-cell responses via nuclear exit of a mechanorepressor. *Nat. Mater.* 14 951–960. 10.1038/nmat4350 26168347 PMC4545733

[B12] Discher D. E. Smith L. Cho S. Colasurdo M. Garcia A. J. Safran S. (2017). Matrix mechanosensing: from scaling concepts in ’omics data to mechanisms in the nucleus, regeneration, and cancer. *Annu. Rev. Biophys.* 46 295–315. 10.1146/annurev-biophys-062215-011206 28532215 PMC5444306

[B13] Dominici M. Le Blanc K. Mueller I. Slaper-Cortenbach I. Marini F. Krause D. (2006). Minimal criteria for defining multipotent mesenchymal stromal cells. The international society for cellular therapy position statement. *Cytotherapy* 8 315–317. 10.1080/14653240600855905 16923606

[B14] Dopie J. Skarp K. P. Rajakyla E. K. Tanhuanpaa K. Vartiainen M. K. (2012). Active maintenance of nuclear actin by importin 9 supports transcription. *Proc. Natl. Acad. Sci. U.S.A.* 109 E544–E552. 22323606 10.1073/pnas.1118880109 PMC3295300

[B15] Downing T. L. Soto J. Morez C. Houssin T. Fritz A. Yuan F. (2013). Biophysical regulation of epigenetic state and cell reprogramming. *Nat. Mater.* 12 1154–1162. 10.1038/nmat3777 24141451 PMC9675045

[B16] Dupont S. Morsut L. Aragona M. Enzo E. Giulitti S. Cordenonsi M. (2011). Role of YAP/TAZ in mechanotransduction. *Nature* 474 179–183. 21654799 10.1038/nature10137

[B17] Engler A. J. Sen S. Sweeney H. L. Discher D. E. (2006). Matrix elasticity directs stem cell lineage specification. *Cell* 126 677–689. 10.1016/j.cell.2006.06.044 16923388

[B18] Fu J. Wang Y. K. Yang M. T. Desai R. A. Yu X. Liu Z. (2010). Mechanical regulation of cell function with geometrically modulated elastomeric substrates. *Nat. Methods* 7 733–736. 10.1038/nmeth.1487 20676108 PMC3069358

[B19] Galipeau J. Krampera M. Barrett J. Dazzi F. Deans R. J. Debruijn J. (2016). International society for cellular therapy perspective on immune functional assays for mesenchymal stromal cells as potency release criterion for advanced phase clinical trials. *Cytotherapy* 18 151–159. 10.1016/j.jcyt.2015.11.008 26724220 PMC4745114

[B20] Gao L. Mcbeath R. Chen C. S. (2010). Stem cell shape regulates a chondrogenic versus myogenic fate through Rac1 and N-cadherin. *Stem Cells* 28 564–572. 20082286 10.1002/stem.308 PMC2896980

[B21] Gerardo H. Lima A. Carvalho J. Ramos J. R. D. Couceiro S. Travasso R. D. M. (2019). Soft culture substrates favor stem-like cellular phenotype and facilitate reprogramming of human mesenchymal stem/stromal cells (hMSCs) through mechanotransduction. *Sci. Rep.* 9:9086. 10.1038/s41598-019-45352-3 PMC6591285 31235788

[B22] Green J. J. Elisseeff J. H. (2016). Mimicking biological functionality with polymers for biomedical applications. *Nature* 540 386–394. 10.1038/nature21005 27974772 PMC8186828

[B23] Gupta M. Sarangi B. R. Deschamps J. Nematbakhsh Y. Callan-Jones A. Margadant F. (2015). Adaptive rheology and ordering of cell cytoskeleton govern matrix rigidity sensing. *Nat. Commun.* 6:7525. 10.1038/ncomms8525 PMC4599139 26109233

[B24] Harris A. R. Jreij P. Fletcher D. A. (2018). Mechanotransduction by the actin cytoskeleton: converting mechanical stimuli into biochemical signals. *Annu. Rev. Biophys.* 47 617–631. 10.1146/annurev-biophys-070816-033547

[B25] Hernandez M. Patzig J. Mayoral S. R. Costa K. D. Chan J. R. Casaccia P. (2016). Mechanostimulation promotes nuclear and epigenetic changes in oligodendrocytes. *J. Neurosci.* 36 806–813. 10.1523/jneurosci.2873-15.2016 26791211 PMC4719016

[B26] Hoch A. I. Leach J. K. (2014). Concise review: optimizing expansion of bone marrow mesenchymal stem/stromal cells for clinical applications. *Stem Cells Transl. Med.* 3 643–652. 10.5966/sctm.2013-0196 24682286 PMC4006491

[B27] Humphries J. D. Wang P. Streuli C. Geiger B. Humphries M. J. Ballestrem C. (2007). Vinculin controls focal adhesion formation by direct interactions with talin and actin. *J. Cell Biol.* 179 1043–1057. 10.1083/jcb.200703036 18056416 PMC2099183

[B28] Jaalouk D. E. Lammerding J. (2009). Mechanotransduction gone awry. *Nat. Rev. Mol. Cell Biol.* 10 63–73. 10.1038/nrm2597 19197333 PMC2668954

[B29] Kanno T. Takahashi T. Tsujisawa T. Ariyoshi W. Nishihara T. (2007). Mechanical stress-mediated Runx2 activation is dependent on Ras/ERK1/2 MAPK signaling in osteoblasts. *J. Cell Biochem.* 101 1266–1277. 10.1002/jcb.21249 17265428

[B30] Kim N. Cho S. G. (2013). Clinical applications of mesenchymal stem cells. *Korea. J. Intern. Med.* 28 387–402. 10.3904/kjim.2013.28.4.387 PMC3712145 23864795

[B31] Klapholz B. Brown N. H. (2017). Talin - the master of integrin adhesions. *J. Cell Sci.* 130 2435–2446. 10.1242/jcs.190991 28701514

[B32] Kuddannaya S. Chuah Y. J. Lee M. H. Menon N. V. Kang Y. Zhang Y. (2013). Surface chemical modification of poly(dimethylsiloxane) for the enhanced adhesion and proliferation of mesenchymal stem cells. *ACS Appl. Mater. Interf.* 5 9777–9784. 10.1021/am402903e 24015724

[B33] Kumar A. Placone J. K. Engler A. J. (2017). Understanding the extracellular forces that determine cell fate and maintenance. *Development* 144 4261–4270. 10.1242/dev.158469 29183939 PMC5769638

[B34] Kureel S. K. Mogha P. Khadpekar A. Kumar V. Joshi R. Das S. (2019). Soft substrate maintains proliferative and adipogenic differentiation potential of human mesenchymal stem cells on long-term expansion by delaying senescence. *Biol. Open* 8:bio039453. 10.1242/bio.039453 31023646 PMC6503999

[B35] Kusuma G. D. Carthew J. Lim R. Frith J. E. (2017). Effect of the microenvironment on mesenchymal stem cell paracrine signaling: opportunities to engineer the therapeutic effect. *Stem Cells Dev.* 26 617–631. 10.1089/scd.2016.0349 28186467

[B36] Lee J. Abdeen A. A. Kilian K. A. (2014). Rewiring mesenchymal stem cell lineage specification by switching the biophysical microenvironment. *Sci. Rep.* 4:5188. 10.1038/srep05188 PMC4046125 24898422

[B37] Leite C. Silva N. T. Mendes S. Ribeiro A. De Faria J. P. Lourenco T. (2014). Differentiation of human umbilical cord matrix mesenchymal stem cells into neural-like progenitor cells and maturation into an oligodendroglial-like lineage. *PLoS One* 9:e111059. 10.1371/journal.pbio.1000059 25357129 PMC4214693

[B38] Lessey E. C. Guilluy C. Burridge K. (2012). From mechanical force to RhoA activation. *Biochemistry* 51 7420–7432. 10.1021/bi300758e 22931484 PMC3567302

[B39] Lima A. F. May G. Diaz-Colunga J. Pedreiro S. Paiva A. Ferreira L. (2018). Osmotic modulation of chromatin impacts on efficiency and kinetics of cell fate modulation. *Sci. Rep.* 8:7210. 10.1038/s41598-018-25517-2 PMC5940679 29740078

[B40] Lourenco T. Paes De Faria J. Bippes C. A. Maia J. Lopes-Da-Silva J. A. (2016). Modulation of oligodendrocyte differentiation and maturation by combined biochemical and mechanical cues. *Sci. Rep.* 6:21563. 10.1038/srep21563 PMC4754901 26879561

[B41] Maekawa M. Ishizaki T. Boku S. Watanabe N. Fujita A. Iwamatsu A. (1999). Signaling from Rho to the actin cytoskeleton through protein kinases ROCK and LIM-kinase. *Science* 285 895–898. 10.1126/science.285.5429.895 10436159

[B42] Mammoto A. Mammoto T. Ingber D. E. (2012). Mechanosensitive mechanisms in transcriptional regulation. *J. Cell Sci.* 125 3061–3073. 10.1242/jcs.093005 22797927 PMC3434847

[B43] Marjoram R. J. Lessey E. C. Burridge K. (2014). Regulation of RhoA activity by adhesion molecules and mechanotransduction. *Curr. Mol. Med.* 14 199–208. 10.2174/1566524014666140128104541 24467208 PMC3929014

[B44] May R. C. (2001). The Arp2/3 complex: a central regulator of the actin cytoskeleton. *Cell. Mol. Life Sci.* 58 1607–1626. 10.1007/pl00000800 11706988 PMC11337294

[B45] Missirlis Y. F. (2016). Mechanoepigenetics. *Front. Cell Dev. Biol.* 4:113. 10.3389/fcell.2016.00113 27790615 PMC5064176

[B46] Mizuno K. (2013). Signaling mechanisms and functional roles of cofilin phosphorylation and dephosphorylation. *Cell Signal.* 25 457–469. 10.1016/j.cellsig.2012.11.001 23153585

[B47] Moore S. W. Roca-Cusachs P. Sheetz M. P. (2010). Stretchy proteins on stretchy substrates: the important elements of integrin-mediated rigidity sensing. *Dev. Cell* 19 194–206. 10.1016/j.devcel.2010.07.018 20708583 PMC5319208

[B48] Morgan J. T. Murphy C. J. Russell P. (2013). What do mechanotransduction, Hippo, Wnt, and TGFbeta have in common? YAP and TAZ as key orchestrating molecules in ocular health and disease. *Exp. Eye Res.* 115 1–12. 10.1016/j.exer.2013.06.012 23792172 PMC3795947

[B49] Munsie L. N. Desmond C. R. Truant R. (2012). Cofilin nuclear-cytoplasmic shuttling affects cofilin-actin rod formation during stress. *J. Cell Sci.* 125 3977–3988. 10.1242/jcs.097667 22623727

[B50] Müller S. Dalgarno K. Dickinson A. Wang X.-N. Nicholson L. (2015). Enhancing the potency of mesenchymal stem cells for tissue regeneration. *Intern. J. Stem Cell Res. Ther.* 2:13.

[B51] Nebl G. Meuer S. C. Samstag Y. (1996). Dephosphorylation of serine 3 regulates nuclear translocation of cofilin. *J. Biol. Chem.* 271 26276–26280. 10.1074/jbc.271.42.26276 8824278

[B52] Ngo K. X. Umeki N. Kijima S. T. Kodera N. Ueno H. Furutani-Umezu N. (2016). Allosteric regulation by cooperative conformational changes of actin filaments drives mutually exclusive binding with cofilin and myosin. *Sci. Rep.* 6:35449. 10.1038/srep35449 PMC5071871 27762277

[B53] Obrdlik A. Percipalle P. (2011). The F-actin severing protein cofilin-1 is required for RNA polymerase II transcription elongation. *Nucleus* 2 72–79. 10.4161/nucl.14508 21647301 PMC3104811

[B54] Ohashi K. (2015). Roles of cofilin in development and its mechanisms of regulation. *Dev. Growth Differ.* 57 275–290. 10.1111/dgd.12213 25864508

[B55] Pendleton A. Pope B. Weeds A. Koffer A. (2003). Latrunculin B or ATP depletion induces cofilin-dependent translocation of actin into nuclei of mast cells. *J. Biol. Chem.* 278 14394–14400. 10.1074/jbc.m206393200 12566455

[B56] Peng T. Liu L. Maclean A. L. Wong C. W. Zhao W. Nie Q. (2017). A mathematical model of mechanotransduction reveals how mechanical memory regulates mesenchymal stem cell fate decisions. *BMC Syst. Biol.* 11:55. 10.1186/s12918-017-0429-x 28511648 PMC5434622

[B57] Percipalle P. (2013). Co-transcriptional nuclear actin dynamics. *Nucleus* 4 43–52. 10.4161/nucl.22798 23138849 PMC3585027

[B58] Pittenger M. F. Mackay A. M. Beck S. C. Jaiswal R. K. Douglas R. Mosca J. D. (1999). Multilineage potential of adult human mesenchymal stem cells. *Science* 284 143–147. 10.1126/science.284.5411.143 10102814

[B59] Provenzano P. P. Keely P. J. (2011). Mechanical signaling through the cytoskeleton regulates cell proliferation by coordinated focal adhesion and Rho GTPase signaling. *J. Cell Sci.* 124 1195–1205. 10.1242/jcs.067009 21444750 PMC3065381

[B60] Prunier C. Prudent R. Kapur R. Sadoul K. LafanechèRe L. (2017). LIM kinases: cofilin and beyond. *Oncotarget* 8 41749–41763. 10.18632/oncotarget.16978 28445157 PMC5522193

[B61] Rosales A. M. Anseth K. S. (2016). The design of reversible hydrogels to capture extracellular matrix dynamics. *Nat. Rev. Mater.* 1:15012. 10.1038/natrevmats.2015.12 PMC5714327 29214058

[B62] Roy B. Venkatachalapathy S. Ratna P. Wang Y. Jokhun D. S. Nagarajan M. (2018). Laterally confined growth of cells induces nuclear reprogramming in the absence of exogenous biochemical factors. *Proc. Natl. Acad. Sci. U.S.A.* 115 E4741–E4750. 29735717 10.1073/pnas.1714770115 PMC6003522

[B63] Shivashankar G. V. (2011). Mechanosignaling to the cell nucleus and gene regulation. *Annu. Rev. Biophys.* 40 361–378. 10.1146/annurev-biophys-042910-155319 21391812

[B64] Shivashankar G. V. (2019). Mechanical regulation of genome architecture and cell-fate decisions. *Curr. Opin. Cell Biol.* 56 115–121. 10.1016/j.ceb.2018.12.001 30554028

[B65] Squillaro T. Peluso G. Galderisi U. (2016). Clinical trials with mesenchymal stem cells: an update. *Cell Transpl.* 25 829–848. 10.3727/096368915x689622 26423725

[B66] Steurer B. Janssens R. C. Geverts B. Geijer M. E. Wienholz F. Theil A. F. (2018). Live-cell analysis of endogenous GFP-RPB1 uncovers rapid turnover of initiating and promoter-paused RNA polymerase II. *Proc. Natl. Acad. Sci. U.S.A.* 115 E4368–E4376. 29632207 10.1073/pnas.1717920115 PMC5948963

[B67] Sun Y. Chen C. S. Fu J. (2012). Forcing stem cells to behave: a biophysical perspective of the cellular microenvironment. *Annu. Rev. Biophys.* 41 519–542. 10.1146/annurev-biophys-042910-155306 22404680 PMC4123632

[B68] Sun Y. Yong K. M. Villa-Diaz L. G. Zhang X. Chen W. Philson R. (2014). Hippo/YAP-mediated rigidity-dependent motor neuron differentiation of human pluripotent stem cells. *Nat. Mater.* 13 599–604. 10.1038/nmat3945 24728461 PMC4051885

[B69] Swift J. Discher D. E. (2014). The nuclear lamina is mechano-responsive to ECM elasticity in mature tissue. *J. Cell Sci.* 127 3005–3015. 10.1242/jcs.149203 24963133 PMC4095853

[B70] Swift J. Ivanovska I. L. Buxboim A. Harada T. Dingal P. C. Pinter J. (2013). Nuclear lamin-A scales with tissue stiffness and enhances matrix-directed differentiation. *Science* 341:1240104. 10.1126/science.1240104 23990565 PMC3976548

[B71] Szentirmay M. N. Sawadogo M. L. (2000). Spatial organization of RNA polymerase II transcription in the nucleus. *Nucleic Acids Res.* 28 2019–2025. 10.1093/nar/28.10.2019 10773068 PMC105382

[B72] Thievessen I. Thompson P. M. Berlemont S. Plevock K. M. Plotnikov S. V. Zemljic-Harpf A. (2013). Vinculin-actin interaction couples actin retrograde flow to focal adhesions, but is dispensable for focal adhesion growth. *J. Cell Biol.* 202 163–177. 10.1083/jcb.201303129 23836933 PMC3704983

[B73] Toh K. C. Ramdas N. M. Shivashankar G. V. (2015). Actin cytoskeleton differentially alters the dynamics of lamin A, HP1alpha and H2B core histone proteins to remodel chromatin condensation state in living cells. *Integr. Biol.* 7 1309–1317. 10.1039/c5ib00027k 26359759

[B74] Trichet L. Le Digabel J. Hawkins R. J. Vedula S. R. Gupta M. Ribrault C. (2012). Evidence of a large-scale mechanosensing mechanism for cellular adaptation to substrate stiffness. *Proc. Natl. Acad. Sci. U.S.A.* 109 6933–6938. 10.1073/pnas.1117810109 22509005 PMC3344951

[B75] Uhler C. Shivashankar G. V. (2017). Chromosome intermingling: mechanical hotspots for genome regulation. *Trends Cell Biol.* 27 810–819. 10.1016/j.tcb.2017.06.005 28728836

[B76] Vining K. H. Mooney D. J. (2017). Mechanical forces direct stem cell behaviour in development and regeneration. *Nat. Rev. Mol. Cell. Biol.* 18 728–742. 10.1038/nrm.2017.108 29115301 PMC5803560

[B77] Vizcaíno J. A. Csordas A. Del-Toro N. Dianes J. A. Griss J. Lavidas I. (2016). 2016 update of the PRIDE database and its related tools. *Nucleic Acids Res.* 44 D447–D456. 26527722 10.1093/nar/gkv1145 PMC4702828

[B78] Vogl A. M. Phu L. Becerra R. Giusti S. A. Verschueren E. Hinkle T. B. (2020). Global site-specific neddylation profiling reveals that NEDDylated cofilin regulates actin dynamics. *Nat. Struct. Mol. Biol.* 27 210–220. 10.1038/s41594-019-0370-3 32015554

[B79] Wagner W. Wein F. Seckinger A. Frankhauser M. Wirkner U. Krause U. (2005). Comparative characteristics of mesenchymal stem cells from human bone marrow, adipose tissue, and umbilical cord blood. *Exp. Hematol.* 33 1402–1416. 10.1016/j.exphem.2005.07.003 16263424

[B80] Wang N. Tytell J. D. Ingber D. E. (2009). Mechanotransduction at a distance: mechanically coupling the extracellular matrix with the nucleus. *Mol. Cell Biol.* 10 75–82. 10.1038/nrm2594 19197334

[B81] Wiggan O. Schroder B. Krapf D. Bamburg J. R. Deluca J. G. (2017). Cofilin regulates nuclear architecture through a myosin-II dependent mechanotransduction module. *Sci. Rep.* 7:40953. 10.1038/srep40953 PMC5244421 28102353

[B82] Wioland H. Guichard B. Senju Y. Myram S. Lappalainen P. Jegou A. (2017). ADF/cofilin accelerates actin dynamics by severing filaments and promoting their depolymerization at both ends. *Curr. Biol.* 27 1956–1967. 28625781 10.1016/j.cub.2017.05.048 PMC5505867

[B83] Yang C. Tibbitt M. W. Basta L. Anseth K. S. (2014). Mechanical memory and dosing influence stem cell fate. *Nat. Mater.* 13 645–652. 10.1038/nmat3889 24633344 PMC4031270

[B84] Yang N. Higuchi O. Ohashi K. Nagata K. Wada A. Kangawa K. (1998). Cofilin phosphorylation by LIM-kinase 1 and its role in Rac-mediated actin reorganization. *Lett. Nat.* 393 809–812. 10.1038/31735 9655398

[B85] Yu L. Li C. M. Liu Y. Gao J. Wang W. Gan Y. (2009). Flow-through functionalized PDMS microfluidic channels with dextran derivative for ELISAs. *Lab. Chip* 9 1243–1247. 19370243 10.1039/b816018j

[B86] Zhao X. H. Laschinger C. Arora P. Szaszi K. Kapus A. Mcculloch C. A. (2007). Force activates smooth muscle alpha-actin promoter activity through the Rho signaling pathway. *J. Cell Sci.* 120 1801–1809. 10.1242/jcs.001586 17456553

