# Supplementary material for: Cofilin-1 Is a Mechanosensitive Regulator of Transcription
Source: Front Cell Dev Biol. 2020 Jul 30;8:678. doi: 10.3389/fcell.2020.00678 (PMC7438942; doi:10.3389/fcell.2020.00678)
Supplement: Supplementary file 1 [file Data_Sheet_1.pdf]

A

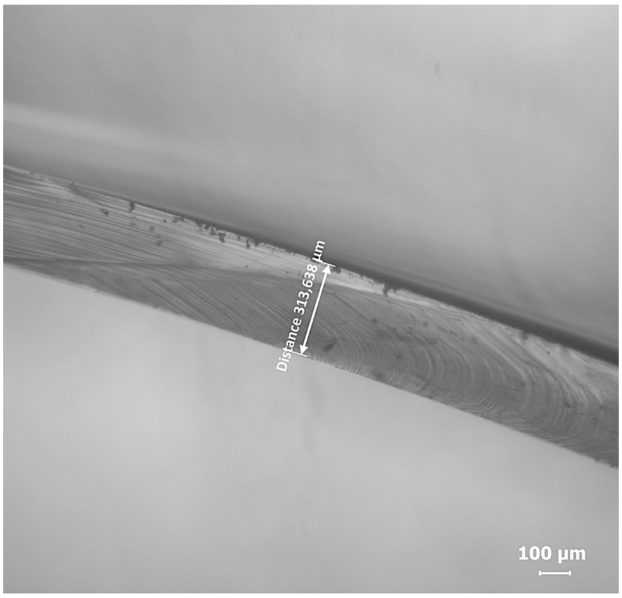

PDMS tickness = 315.5 ± 4.6 µm (n=5)

B

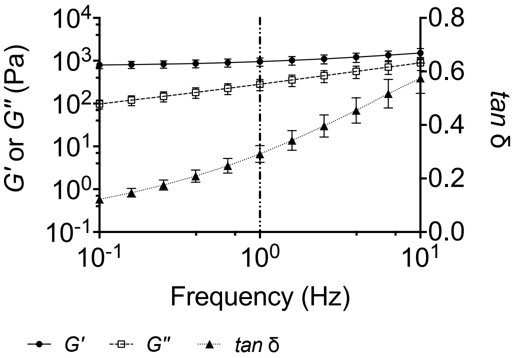

C

Values are expressed as mean ± SEM			
G' (1 Hz)	*G"* (1 Hz)	*E* (1 Hz)	PDMS thickness
957 ± 208 Pa	284 ± 83 Pa	2870 ± 625 Pa	315.5 ± 4.6 µm

Figure S1 – Characterization of PDMS substrates. **(A)** Measurement of PDMS substrate thickness. Representative phase-contrast microscopy image of a 10:1 PDMS substrate sliced on the central zone and the respective thickness measurement. This procedure was performed using 5 independent batches and is indicated as mean ± SEM. **(B)** Rheological measurement of storage modulus (G´), loss modulus (G´´) and tan δ by rehometry of 40:1 PDMS substrates across a frequency sweep between 0.1 and 10 Hz with 1% strain at 37°C. Data represents mean ± SEM obtained from substrates from 3 independent batches. **(C)** Summary of the values obtained for 40:1 PDMS characterization; values of G´ (1 Hz), G´´(1 Hz), E (Young´s modulus, at 1 Hz) and PDMS thickness are expressed as mean ± SEM of at least three independent experiments.

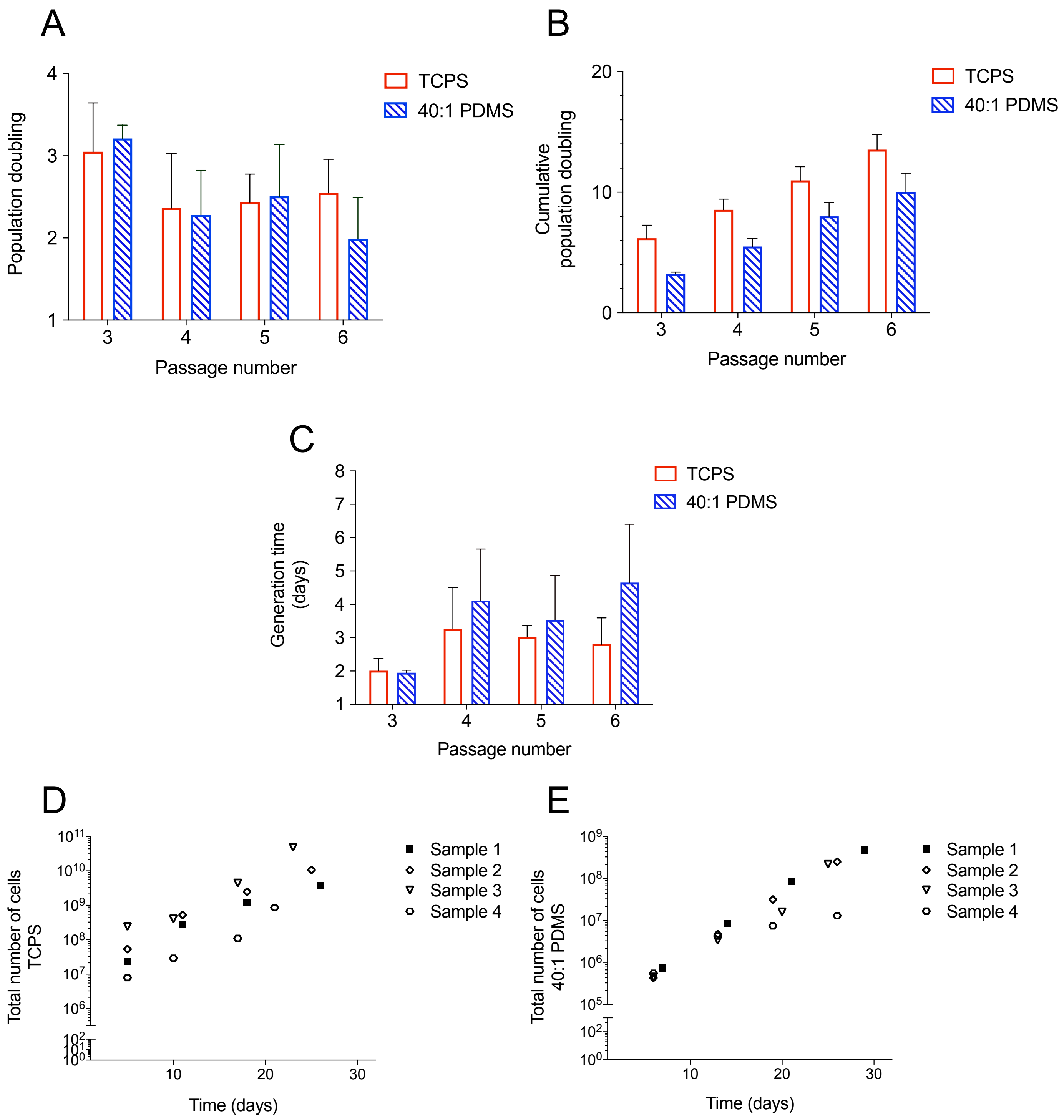

Figure S2 – Proliferation kinetics of hUCM-MSCs cultured on TCPS and 40:1 PDMS. Cells were passaged (from P3 until P6) and counted once they reached a confluence of 80%. **(A)** population doubling. **(B)** cumulative population doubling determined by adding the calculated population doubling to the population doubling of the previous passages. **(C)** generation time (average time between two cell doublings). **(D)** and **(E)** the total number of cells determined over time for both substrates (TCPS and 40:1 PDMS). Bars represent mean ± SEM of at least three independent experiments using cells obtained from different donors. Statistical analysis was performed using the non-parametric Mann-Whitney test with no significant differences found ($p>0.05$).

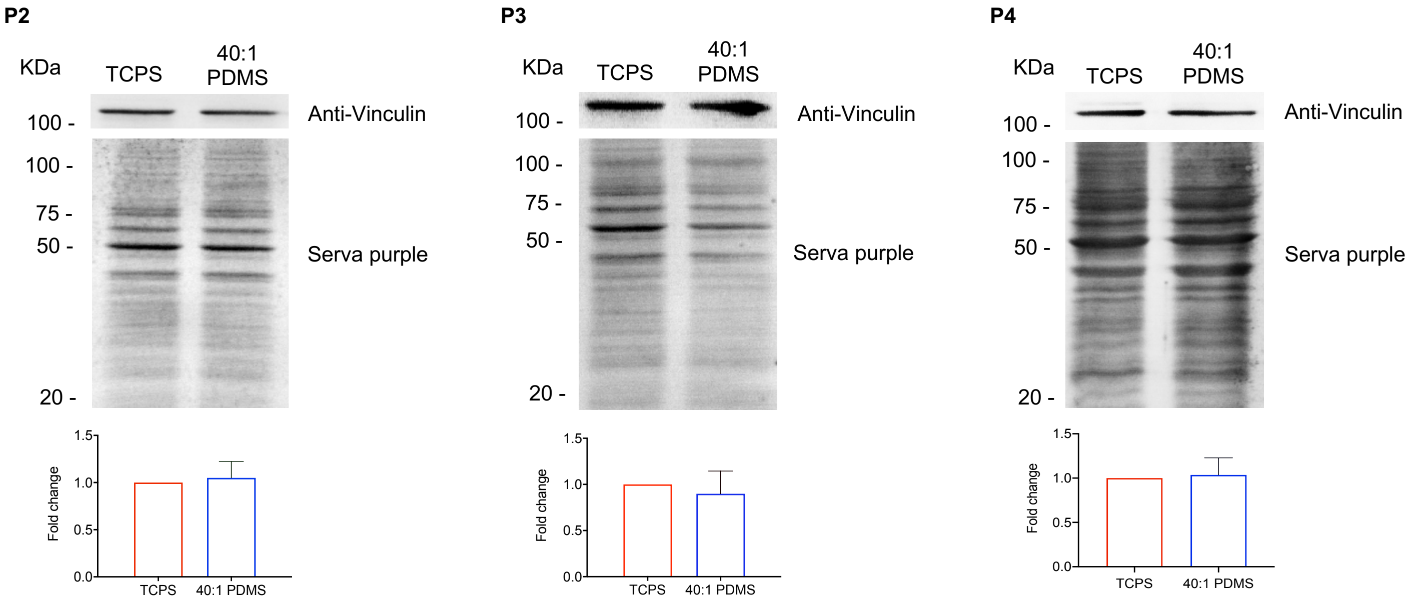

Figure S3 – Western blot analysis (top) of vinculin present in whole-cell protein extracts (separated by SDS-PAGE) obtained from hUCM-MSCs isolated and cultured on TCPS until P1 and then cultured on stiff TCPS or soft 40:1 PDMS until P2, P3 or P4 (as indicated). For quantification analysis (bottom), vinculin expression was normalized using the respective total protein level assessed by staining the WB membrane using SERVA purple (top). Bars (bottom) represent mean ± SEM of three independent experiments. Statistical analysis was performed using a One-Sample t-test (theoretical mean of 1.0), significant differences were not found ($p>0.05$).

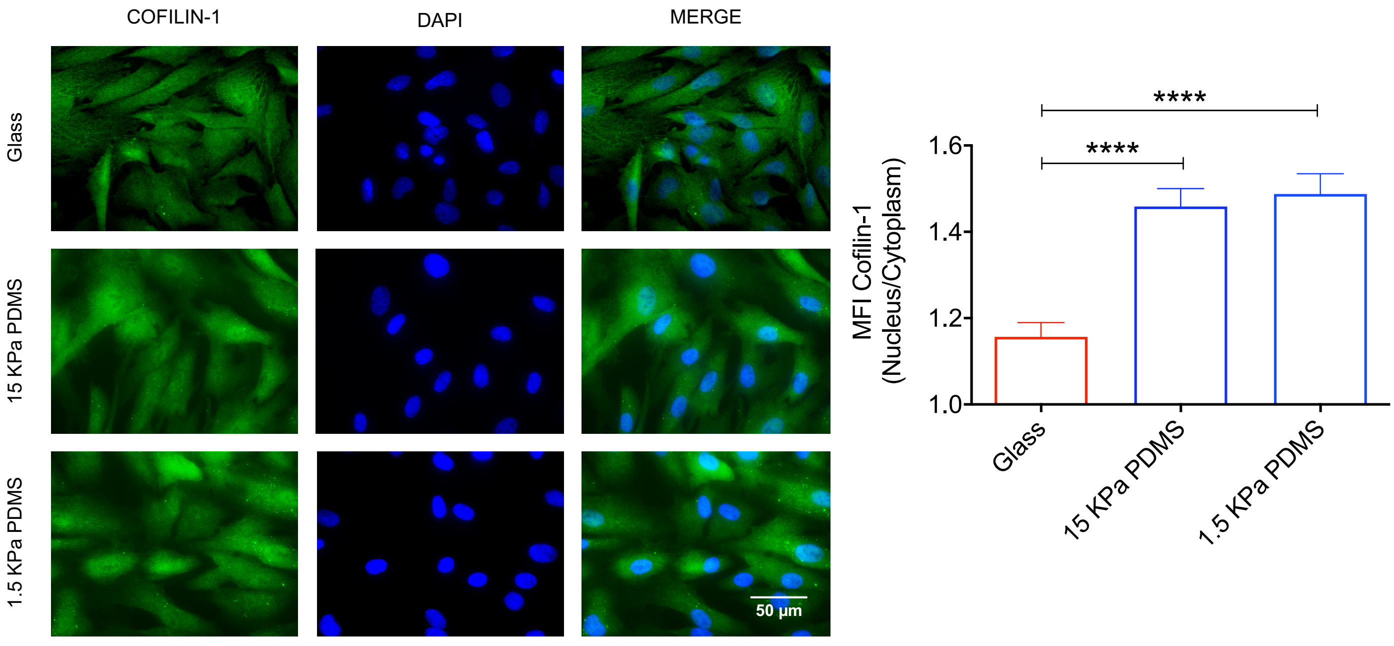

Figure S4 – hUCM-MSCs respond to distinct substrate stiffness. Representative fluorescence microscopy images (left) and respective MFI quantification of Cofilin-1 nucleus/cytoplasm ratio present in cells cultured on glass coverslips, and commercially available 15 KPa or 1.5 KPa PDMS. hUCM-MSCs were fixed and stained with anti-Cofilin-1 antibody (green) and DAPI for nuclear counterstaining (blue). Bars represent mean ± SEM of three independent experiments. Statistical analysis was performed using One-Way ANOVA followed by Dunnett's multiple comparisons test between all conditions (****$p<0.0001$).

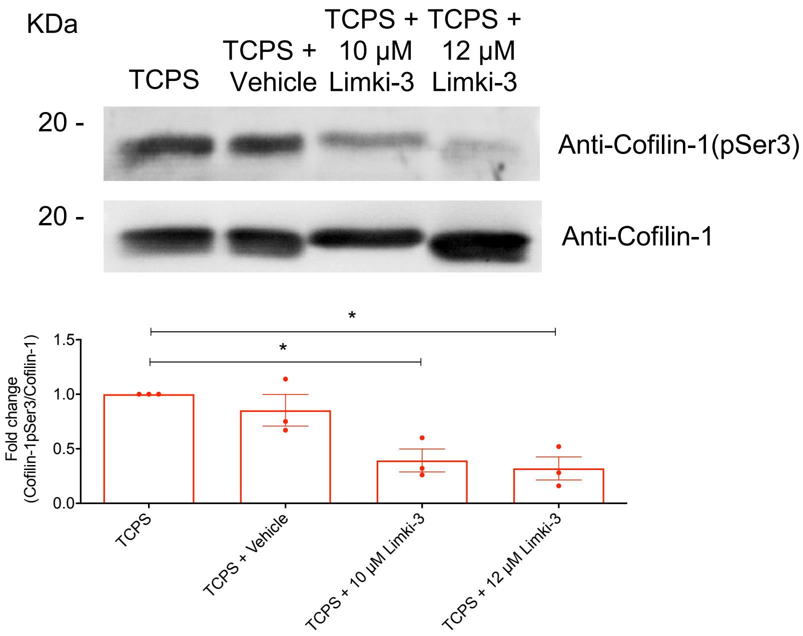

Figure S5 – Western blot analysis (top) of Cofilin-1(pSer3) and Cofilin-1 present in whole-cell protein extracts (separated by SDS-PAGE) obtained from hUCM-MSCs isolated and cultured on stiff TCPS at P2. After 24 h of seeding, cells were incubated with 10 µM or 12 µM of LIM Kinase inhibitor (Limki-3) for an additional 24 h. For quantification analysis (bottom), Cofilin-1(pSer3) expression levels were normalized for the total levels of Cofilin-1 (top). Bars (bottom) represent mean ± SEM of three independent experiments. Statistical analysis was performed using a One-Sample t-test (theoretical mean of 1.0), with significant differences indicated as $^*p<0.05$.

A

(80.1% coverage with 95% confidence)

MASGVAVSDGVIKVFNDMKVRKSSTPEEVKKRKKAVLFCLSEDKKNIILEEGKEILVGD
VGQTVDDPYATFVKMLPDKDCRYALYDATYETKESKKEDLVFIFWAPESAPLKSKMIY
ASSKDAIKKKLTGIKHELQANCYEEVKDRCTLAEKLGGSAVISLEGKPL

B

(87.4% coverage with 95% confidence)

MASGVAVSDGVIKVFNDMKVRKSSTPEEVKKRKKAVLFCLSEDKKNIILEEGKEILVGD
VGQTVDDPYATFVKMLPDKDCRYALYDATYETKESKKEDLVFIFWAPESAPLKSKMIY
ASSKDAIKKKLTGIKHELQANCYEEVKDRCTLAEKLGGSAVISLEGKPL

C

Search with biological modifications & special focus in phosphorylation:

Peptide Evidence									
Peptide Locus	Conf	Sequence	Modifications	Cleavages	ΔMass	Obs MW	z	Sc	Best Spectrum
12299.001	99	ASGVAVSDGVIK	Phospho(S)@2	cleaved M-A@N-term	0.0099	1181.5792	2	8	13.1.1.366.35
12301.001	99	ASGVAVSDGVIK	Acetyl@N-term Phospho(S)@2	cleaved M-A@N-term	0.0056	1223.5854	2	8	14.1.1.423.20

Search with biological modifications without special focus in phosphorylation:

Peptide Evidence									
Peptide Locus	Conf	Sequence	Modifications	Cleavages	ΔMass	Obs MW	z	Sc	Best Spectrum
12181.001	99	ASGVAVSDGVIK	Acetyl@N-term Phospho(S)@2	cleaved M-A@N-term	0.0056	1223.5854	2	8	14.1.1.423.20

D

	PDMS/TCPS		
Fraction	Cofilin-1 (pSer3) levels (median value; n=3)	Cofilin-1 total levels (average; n=3)	Cofilin-1 (pSer3)/Cofilin-1 total levels (median value; n=3)
Soluble fraction	0.35	0.96	0.40

Figure S6 – Mass spectrometry evidence of a reduction in phospho-Cofilin-1 in hUCM-MSCs cultured in soft substrates. **(A)** Sequence coverage of Cofilin-1 achieved in a protein search avoiding the inclusion of biological modifications. **(B)** Sequence coverage (top) of Cofilin-1 achieved in protein searches focused on biological modifications. Color code indicates peptides confidence: green residues correspond to peptides identified with 95% confidence; yellow for peptides with confidence between 50 and 95%; and grey corresponds to unidentified residues. **(C)** List of modifications of the peptide "ASGVAVSDGVIK" identified with confidence (>95%). **(D)** Fold change values of Phospho (Ser3)- and total- Cofilin-1 in hUCM-MSCs cultured in PDMS in comparison with TCPS and indication of the median ratio of Cofilin-1 (pSer3)/total Cofilin-1. Phospho (Ser3)-Cofilin-1 relative levels were determined by the "semi-targeted" analysis of the "AS[Phos]GVAVSDGVIK" peptide using the SWATH data previously acquired.

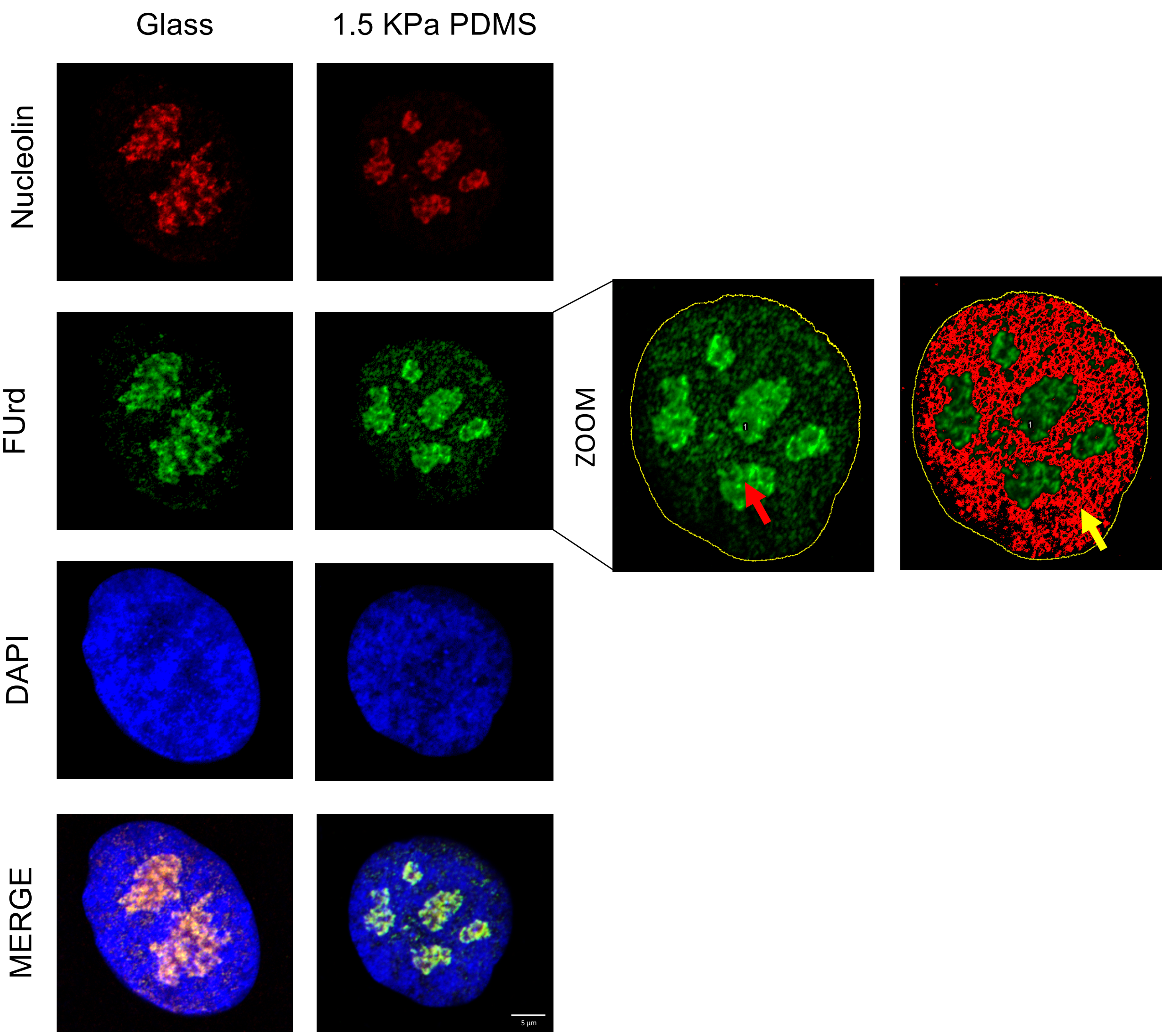

Figure S7 – Representative confocal microscopy images of the nuclei of hUCM-MSCs cultured on glass coverslips or 1.5 KPa PDMS. Cells were incubated with FUrd for 45 min, fixed and stained with an anti-Nucleolin antibody (red), anti-BrdU (green) and DAPI for nuclear counterstaining (blue). On the right, zoomed images of FUrd with the red arrow pointing to nucleolar foci (left) and the yellow arrow to nucleoplasmic foci (right).

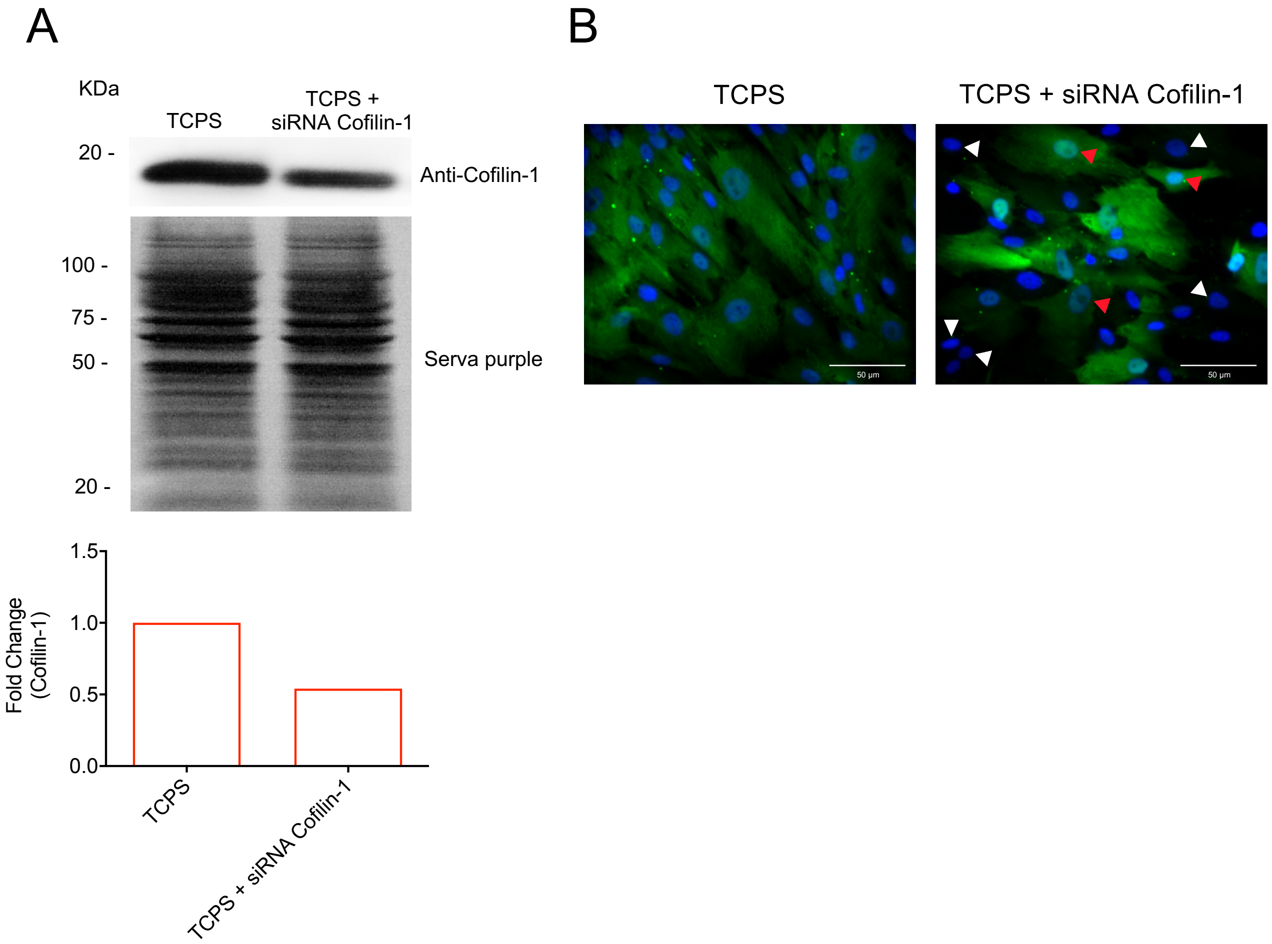

Figure S8 – Cofilin-1 gene silencing by siRNA transfection. **(A)** western blot analysis (top) of Cofilin-1 present in whole-cell protein extracts (separated by SDS-PAGE) obtained from hUCM-MSCs cultured and transfected or not on TCPS at P2. Cells were transfected according to the manufacturer's instructions using lipofectamine 3000 with 100 nM of SignalSilence® Cofilin siRNA I. 72 h after transfection cells were detached and whole-cell protein extracts analysed by western blot. For western blot quantification (bottom), Cofilin-1 expression was normalized using the respective total protein level assessed by staining the WB membrane using SERVA purple (top). Bars (bottom) represent the absolute value obtained for one single experiment. **(B)** representative fluorescence microscopy images of cells cultured and transfected or not on TCPS at P2. After transfection, cells were stained with anti-Cofilin-1 antibody (green) and DAPI for nuclear counterstaining (blue). White arrowheads indicate cells in which the knock-down was effective, and the red arrowheads indicate cells in which it was not.

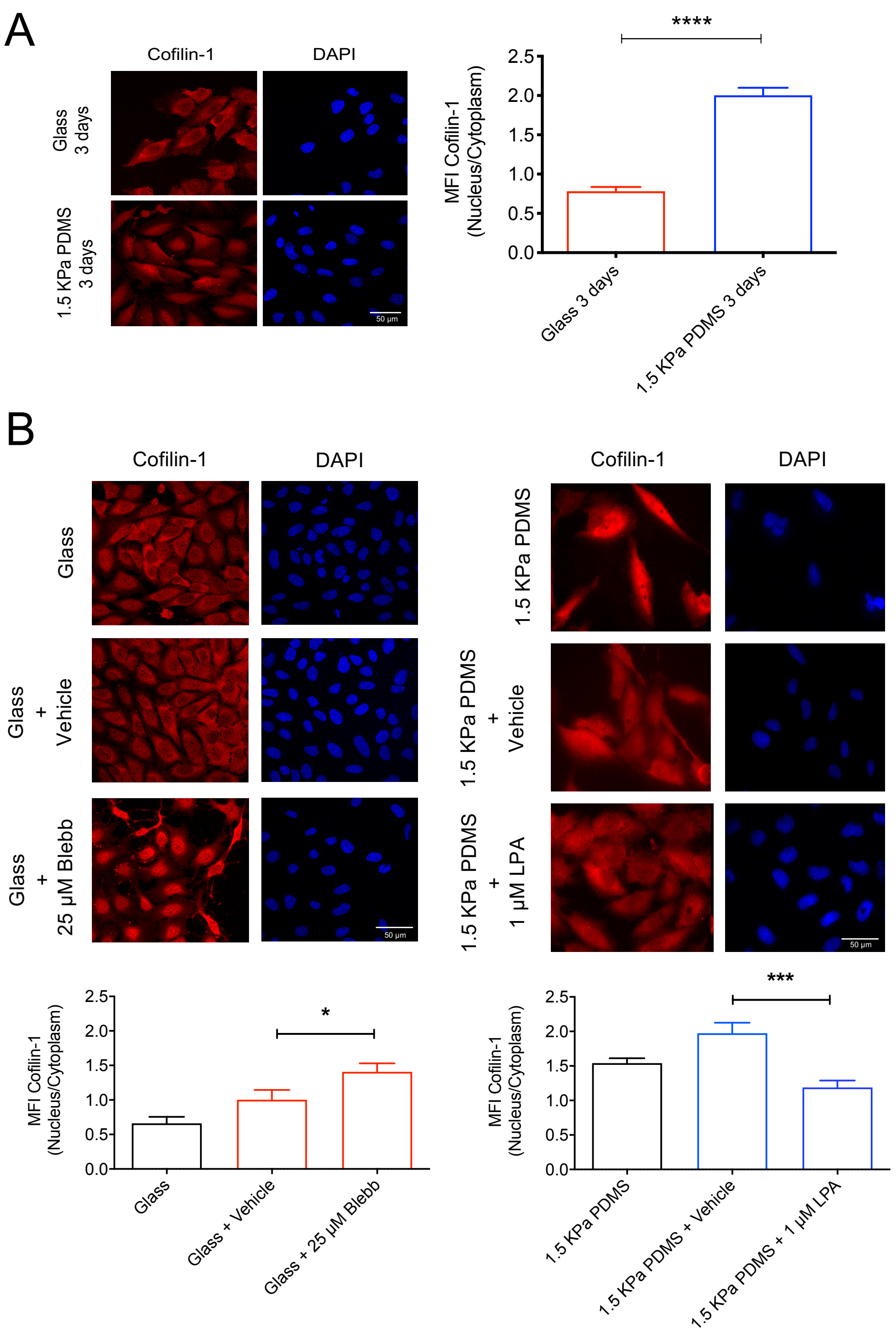

Figure S9 – MRC-5 cells respond to substrate stiffness and soluble modulators of actomyosin contractility. **(A)** representative fluorescence microscopy images (left) and respective MFI quantification of Cofilin-1 nucleus/cytoplasm ratio present in cells cultured during 3 days on glass coverslips or 1.5 KPa PDMS. MRC-5 cells were fixed and stained with anti-Cofilin-1 antibody (red) and DAPI for nuclear counterstaining (blue). Bars represent mean ± SEM of three independent experiments. Statistical analysis was performed using a non-parametric Mann-Whitney test with significant differences represented as ****$p<0.0001$. **(B)** representative fluorescence microscopy images and respective MFI quantification of Cofilin-1 nucleus/cytoplasm ratio for cells cultured on glass coverslips or 1.5 KPa PDMS after treatment with Blebbistatin or LPA. Cells seeded on stiff glass coverslips (left) were cultured for 32h and then incubated or not with Blebbistatin (25 µM) for an additional 40h; cells seeded on soft 1.5 KPa PDMS (right) were cultured for 70h and then incubated or not with LPA (1 µM) for an additional 2h. In both cases, cells were fixed after 72h in culture and stained with anti-Cofilin-1 antibody (red) and DAPI for nuclear counterstaining (blue). Bars represent mean ± SEM of three independent experiments. Statistical analysis was performed using a non-parametric Mann-Whitney test comparing Blebbistatin or LPA against the corresponding vehicle condition. Significant differences are indicated as *$p<0.05$ and ***$p<0.001$.

Table S1 – Panel of monoclonal antibodies reagents (with clones and commercial source) used for immunophenotypic characterization of hUCM-MSCs by flow cytometry.

	Fluorochrome				
Tube	V450	V500-C	PE	PerCPCy5.5	APC
1	HLA-DR (L243) BD	CD45 (2D1) BD	CD105 (TEA3/17.1.1) Beckman Coulter	CD34 (8G12) BD	CD10 (HI10α) BD
2	HLA-DR (L243) BD	CD45 (2D1) BD	CD13 (clone L138) BD	CD34 (8G12) BD	CD10 (HI10α) BD
3	HLA-DR (L243) BD	CD45 (2D1) BD	CD90 PE (5E10) BD Pharmingen	CD34 (8G12) BD	CD10 (HI10α) BD

Abbreviations: PE, phycoerythrin; PerCPCy5.5, peridinin chlorophyll protein-cyanine 5.5; APC, allophycocyanin. Commercial sources: BD (Becton Dickinson Biosciences, San Jose, CA, USA); Beckman Coulter (Miami, FL, USA); BD Pharmingen (San Diego, CA, USA).

Table S2 – List of antibodies and dyes used for western blot and immunocytochemistry.

Antibodies & Dyes	Dilution & Concentration (μg/ml)		Provider	Catalogue number
	Western Blot	Immunocytochemistry		
Mouse anti-Vinculin	1:5000	–	Abcam	ab1194
Rabbit anti-Cofilin	1:2000	1:600	Cell Signalling	D3F9
Rabbit anti-Cofilin-1 (pSer3)	1:1000	–	Thermo Fisher Scientific	44-1072G
Mouse anti-BrdU (clone BU-33)	–	1:500	Sigma-Aldrich	B8434
Mouse anti-Nucleolin [364-5]	–	1:1000	Abcam	ab136649
Alexa Fluor 488 goat anti-rabbit	–	1:500	Life Technologies	A11008
Alexa Fluor 568 goat anti-rabbit	–	1:200	Life Technologies	A11036
Alexa Fluor 568 goat anti-mouse	–	1:200	Life Technologies	A11031
Alexa Flour 488 goat anti-mouse	–	1:200	Life Technologies	A11001
TRITC-labelled Phalloidin	–	1:200	Enzo Life Sciences	R415
DAPI	–	0.8	Molecular Probes-Life Technologies	D3571

Table S3 – Summary of the values obtained for the proteomics analysis of P4 hUCM-MSCs cultured on 40:1 PDMS in comparison with TCPS; fold change (expressed as mean), standard deviation (SD), coefficient of variation (CV, expressed as percentage), and p-value for Cofilin-1 identified in the membrane fraction and vinculin in the soluble fraction.

Accession number	Protein name	Fraction	Fold change (mean)	SD	% CV	p-value
P23528	Cofilin-1	Membrane fraction	1.295	0.060	4.669	0.014
P18206	Vinculin	Soluble fraction	0.866	0.075	8.643	0.090
