# Supplementary material for: Cofilin-1 Is a Mechanosensitive Regulator of Transcription
Source: Front Cell Dev Biol. 2020 Jul 30;8:678. doi: 10.3389/fcell.2020.00678 (PMC7438942; doi:10.3389/fcell.2020.00678)
Supplement: Supplementary file 2 [file Data_Sheet_2.DOCX]

Supplementary Material

# Supplementary Methods

## SWATH Acquisition

Samples were analyzed on a Triple TOF^TM^ 5600 System (ABSciex®) in two phases: information-dependent acquisition (IDA) was followed by SWATH (Sequential Windowed data independent Acquisition of the Total High-resolution Mass Spectra) acquisition on the same sample. Peptides were resolved by liquid chromatography (nanoLC Ultra 2D, Eksigent®) on a MicroLC column ChromXP^TM^ C18CL (300 μm ID × 15cm length, 3 μm particles, 120 Å pore size, Eksigent®) at 5μL/min. Peptides were eluted into the mass spectrometer with an acetonitrile gradient in 0.1% FA (5% to 35% ACN, in a linear gradient for 45 min), using an electrospray ionization source (DuoSpray^TM^Source, ABSciex®).

For information dependent acquisition (IDA) experiments were performed for each 3 peptides mixtures per samples. The mass spectrometer was set to scanning full spectra (350-1250 m/z) for 250ms, followed by up to 100 MS/MS scans (100–1500 m/z from a dynamic accumulation time – minimum 30 ms for precursor above the intensity threshold of 1000 – in order to maintain a cycle time of 3.3 s). Candidate ions with a charge state between +2 and +5 and counts above a minimum threshold of 10 counts per second were isolated for fragmentation and one MS/MS spectra was collected before adding those ions to the exclusion list for 25 seconds (mass spectrometer operated by Analyst® TF 1.6, ABSciex®). Rolling collision was used with a collision energy spread of 5.

The 3 peptide mixtures of each sample were combined and concentrated, and a single analysis of each sample was set for quantitative analysis by acquisition in SWATH mode. The SWATH setup was essentially as described by Anjo *et al.* (Anjo et al., 2015). The mass spectrometer was operated in a looped product ion mode, and specifically tuned to allow a quadrupole resolution of 25 m/z mass selection. Using an isolation width of 26 m/z (containing 1 m/z for the window overlap), a set of 30 overlapping windows was constructed, covering the precursor mass range of 350–1100 m/z. A 50 ms survey scan (350-1500 m/z) was acquired at the beginning of each cycle, and SWATH-MS/MS spectra were collected from 100-1500 m/z for 100 ms resulting in a cycle time of 3.1 s. Collision energy for each window was determined according to the calculation for a charge +2 ion-centered upon the window with a collision energy spread of 15.

**References**

Anjo, S.I., Santa, C., and Manadas, B. (2015). Short GeLC-SWATH: a fast and reliable quantitative approach for proteomic screenings. *Proteomics* 15**,** 757-762. doi: 10.1002/pmic.201400221.

**
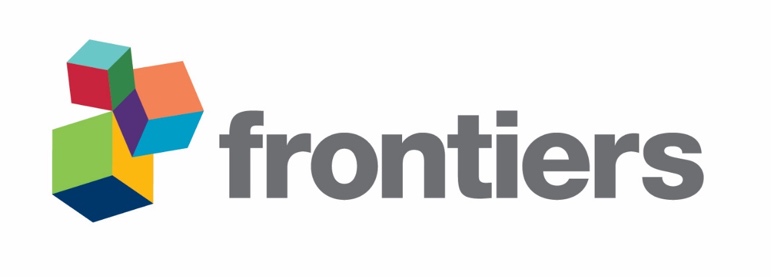
**
